# On the Initialization of Swarm Intelligence Algorithms for Vector Quantization Codebook Design

**DOI:** 10.3390/s24082606

**Published:** 2024-04-19

**Authors:** Verusca Severo, Felipe B. S. Ferreira, Rodrigo Spencer, Arthur Nascimento, Francisco Madeiro

**Affiliations:** 1 Polytechnic School of Pernambuco, University of Pernambuco, Recife 50720-001, Brazil; rshc@poli.br (R.S.); artn@poli.br (A.N.); madeiro@poli.br (F.M.); 2 Engineering Campus, Rural Federal University of Pernambuco, Cabo de Santo Agostinho 54518-430, Brazil; felipe.bsferreira@ufrpe.br

**Keywords:** vector quantization, image compression, initialization, swarm intelligence

## Abstract

Vector Quantization (VQ) is a technique with a wide range of applications. For example, it can be used for image compression. The codebook design for VQ has great significance in the quality of the quantized signals and can benefit from the use of swarm intelligence. Initialization of the Linde–Buzo–Gray (LBG) algorithm, which is the most popular VQ codebook design algorithm, is a step that directly influences VQ performance, as the convergence speed and codebook quality depend on the initial codebook. A widely used initialization alternative is random initialization, in which the initial set of codevectors is drawn randomly from the training set. Other initialization methods can lead to a better quality of the designed codebooks. The present work evaluates the impacts of initialization strategies on swarm intelligence algorithms for codebook design in terms of the quality of the designed codebooks, assessed by the quality of the reconstructed images, and in terms of the convergence speed, evaluated by the number of iterations. Initialization strategies consist of a combination of codebooks obtained by initialization algorithms from the literature with codebooks composed of vectors randomly selected from the training set. The possibility of combining different initialization techniques provides new perspectives in the search for the quality of the VQ codebooks. Nine initialization strategies are presented, which are compared with random initialization. Initialization strategies are evaluated on the following algorithms for codebook design based on swarm clustering: modified firefly algorithm—Linde–Buzo–Gray (M-FA-LBG), modified particle swarm optimization—Linde–Buzo–Gray (M-PSO-LBG), modified fish school search—Linde–Buzo–Gray (M-FSS-LBG) and their accelerated versions (M-FA-LBGa, M-PSO-LBGa and M-FSS-LBGa) which are obtained by replacing the LBG with the accelerated LBG algorithm. The simulation results point out to the benefits of the proposed initialization strategies. The results show gains up to 4.43 dB in terms of PSNR for image Clock with M-PSO-LBG codebooks of size 512 and codebook design time savings up to 67.05% for image Clock, with M-FF-LBGa codebooks with size N=512, by using initialization strategies in substitution to Random initialization.

## 1. Introduction

The diversity of signals transmitted on the network (audio, video, image and voice), the number of connected devices and the wide use of artificial intelligence in virtual assistants, smartphones, tablets and computers promote modernization of everyday actions and lead to demand for efficient data traffic and digital signal processing systems [1,2].

In this scenario, signal compression techniques become increasingly necessary. Compression techniques are predominantly divided into two groups: lossless compression, widely used in medical images, and lossy compression. In lossy compression it is generally possible to obtain higher compression rates [3].

Vector quantization (VQ) has been extensively used in lossy compression techniques. VQ consists of mapping a sequence of *K*-dimensional input vectors by a corresponding sequence of *K*-dimensional reconstruction vectors of a finite subset called a codebook [4]. The quality of VQ-based systems is directly associated with the quality of the codebooks. For this reason, codebook design is a relevant problem in the context of VQ.

The most used method in designing codebooks for VQ is the LBG algorithm, proposed by Linde, Buzo and Gray. LBG [5] starts with a codebook that is iteratively improved. The initial codebook has a large impact on both the convergence speed of the algorithm and the quality of the reconstructed signals.

In recent years, a new family of algorithms has emerged from combining swarm intelligence with LBG [6,7,8]. The versatility of swarm techniques allows them to be combined with the LBG algorithm and used in codebook design.

The objective of this paper is to evaluate the use of initialization strategies [9,10,11,12,13,14] in LBG + swarm intelligence algorithms for QV codebook design with regard to the quality of the reconstructed images and the convergence speed of the algorithms (number of iterations). The possibility of combining different initialization techniques provides new perspectives in the search for quality VQ codebooks.

Thus, we can highlight the main contributions of this work:

- The evaluation of a variety of combinations of initialization techniques for image VQ codebook design by swarm intelligence-based algorithms, in terms of the average peak signal-to-noise ratio (PSNR) of the reconstructed images and in terms of the average number of iterations of these algorithms.
- The evaluation of the influence of a variety of combinations of initialization techniques (precisely, nine initialization strategies) in swarm-based VQ codebook algorithms (Particle Swarm Optimization, Firefly Algorithm and Fish School Search) considering two distinct scenarios—when the algorithms are combined with the conventional LBG algorithm and when they are combined with an accelerated version of the LBG algorithm—by using statistical instruments that allow one to group algorithms that do not present statistically significant differences in performance.

The remainder of the article is organized as follows. Section 2 presents the fundamentals of VQ and the description of the LBG algorithm and its accelerated version. The initialization strategies are described in Section 3. The swarm intelligence-based codebook design techniques used in this paper are presented in Section 4. The methodology used in the experimental setup is presented in Section 5. The results and conclusions are presented in Section 6 and Section 7, respectively.

## 2. Vector Quantization

Efficient coding of signals is an essential process in several areas and applications, such as mobile communications, streaming services and image storage, among others. VQ is an efficient coding technique that aims to reduce the number of bits required to represent a signal [4,15,16,17].

VQ is an extension of scalar quantization to a *K*-dimensional Euclidean space, $R^{K}$, in which each of the *M* vectors of an input signal $X=\{{\vec{x}}_{m},m=1,2,\ldots ,M\}$ is represented by one of *N* vectors (usually with N<<M) belonging to a finite subset of $R^{K}$, $Y=\{{\vec{y}}_{n},n=1,2,\ldots ,N\}$, called a codebook [4,18].

The mapping of vectors from *X* to *Y* occurs through a similarity rule that seeks to reduce the distortion introduced at the end of the vector quantization process. Typically, a widely used measure to evaluate the similarity of vectors is the squared Euclidean distance, given by

(1) $$d\left({\vec{x}}_{m},{\vec{y}}_{n}\right)=\sum_{j=1}^{K}{\left(x_{mj}-y_{nj}\right)}^{2},$$

in which $x_{mj}$ is the *j*-th component of ${\vec{x}}_{m}$ and $y_{nj}$ is the *j*-th component of the vector ${\vec{y}}_{n}$.

Through mapping, the input signal *X* is partitioned into *N* cells $S_{i}=1,2,\ldots ,N$, called Voronois regions, such that

(2) $$S_{i}=\left\{{\vec{x}}_{m}\;:\;d\left({\vec{x}}_{m},\vec{y_{i}}\right)<d\left({\vec{x}}_{m},\vec{y_{o}}\right)\;\forall \;\;o\neq i\right\},$$

in which all input signal vectors belonging to the $S_{i}$ Voronoi region are represented by the codevector ${\vec{y}}_{i}$. This representation leads to an error called distortion, calculated by

(3) $$D=\sum_{i=1}^{N}\sum_{{\vec{x}}_{m}\in S_{i}}d\left({\vec{x}}_{m},{\vec{y}}_{i}\right).$$

Each codevector ${\vec{y}}_{i}$ is represented by a binary codeword $b_{i}$ of length *n* bits. The number of bits required to represent each codevector is $b=\log_{2}N$. Therefore, the code rate of the vector quantizer, which measures the number of bits by vector component and is expressed in bits per sample in voice waveform coding, or in bits per pixel (bpp) in the case of image coding, is given by

(4) $$R=\frac{1}{K}\log_{2}N.$$

An important issue regarding VQ is the compromise between rate and distortion.

The signal reconstructed by VQ is a degraded version of the original signal. Therefore, in order for the VQ-based signal processing systems to perform well, it is necessary to have a good codebook that minimizes the average distortion introduced by representing the input vectors ${\vec{x}}_{m}$ by the corresponding codevectors, satisfying the nearest neighbor condition.

The study of techniques for generating a more representative codebook is a widely explored research topic [19,20,21,22,23,24,25]. Among the various codebook design techniques, the LBG (Linde, Buzo and Gray) algorithm stands out for being the most popular.

### 2.1. LBG Algorithm

LBG [5] is an algorithm proposed by Linde, Buzo and Gray as a generalization of Lloyd’s algorithm for a multidimensional space. LBG corresponds to a clustering algorithm, which starts with an initial solution that is iteratively improved to satisfy the centroid and nearest neighbor conditions.

LBG starts with a codebook, usually composed of vectors randomly obtained from the training set, for example $Z=\{{\vec{z}}_{q},q=1,2,\ldots ,Q\}$, with $\vec{z_{q}}=\left\{z_{q1},z_{q2},\ldots ,z_{qK}\right\}$, which is updated at each iteration based on the calculation of the centroids of each Voronoi region. The calculation of centroids is given by

(5) $$y_{ij}=\frac{1}{Q_{i}}\sum_{{\vec{z}}_{q}\in S_{i}}z_{qj},$$

in which $Q_{i}$ is the number of training vectors in the region $S_{i}$ and $z_{qj}$ is the *j*-th component of ${\vec{z}}_{q}$.

The stopping criterion of the LBG algorithm is based on the calculation of the distortion defined in Equation (3). Distortion is monitored at each iteration and decreases monotonically. The stopping criterion is reached, ending the process, when the percentual distortion variation decrease in the current iteration with respect to the previous one is below a distortion threshold, that is

(6) $$\frac{D_{t-1}-D_{t}}{D_{t}}\leq \epsilon ,$$

in which ϵ corresponds to the distortion threshold and $D_{t}$ corresponds to the distortion calculation in the *t*-th iteration.

LBG can be summarized in the following sequence of steps:

- Step 1 (initialization): set $Y_{0}$ as the initial codebook, set t=0 and the distortion as $D_{-1}=\infty$ (a very large number).
- Step 2 (partitioning): let $Y_{t}$ be the codebook in the *t*-th iteration, allocate each training vector ${\vec{z}}_{q}$ in the Voronoi region according to Equation (2).
- Step 3 (distortion calculation): calculate the total distortion, according to Equation (3).
- Step 4 (stop criterion): If the stopping criterion is satisfied according to Equation (6), the algorithm stops and returns $Y_{t}$ representing the final codebook; otherwise, continue;
- Step 5 (updating codevectors): calculate the new centroids of each Voronoi region according to Equation (5). Let t=t+1 and return to Step 2.

### 2.2. Accelerated LBG Algorithm

Lee et al. proposed in [26] an alternative to accelerate the LBG algorithm, by the use of a scale factor in the step of updating the codevectors.

**Figure 1:**
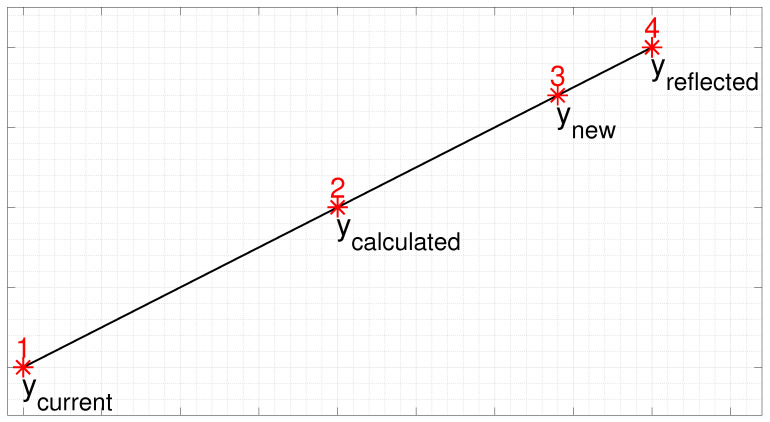
Update of a codevector by the LBGa algorithm with K=2.

In the proposed technique, referred to here as accelerated LBG algorithm (LBGa), initially the codevector is calculated as the centroid of the corresponding Voronoi region, according to Equation (5), and then the new codevector will be one of the points in a line segment that connects the calculated codevector, $y_{\mathrm{calculated}}$, to the reflected point, $y_{\mathrm{reflected}}$ (which corresponds to a reflection of the current codevector relative to the centroid conventionally calculated, $y_{\mathrm{calculated}}$), as illustrated in Figure 1.

Point 2 shown in Figure 1, $y_{\mathrm{calculated}}$, represents, in the LBG algorithm, the new codevector (new centroid of a given Voronoi region). Lee et al. proposed in [26] to choose as a new codevector a point between points 2 and 4 of Figure 1, as follows:

(7) $$y_{\mathrm{new}}=y_{\mathrm{current}}+s\cdot \left(y_{\mathrm{calculated}}-y_{\mathrm{current}}\right),$$

in which *s* corresponds to the scale factor.

**Figure 2:**
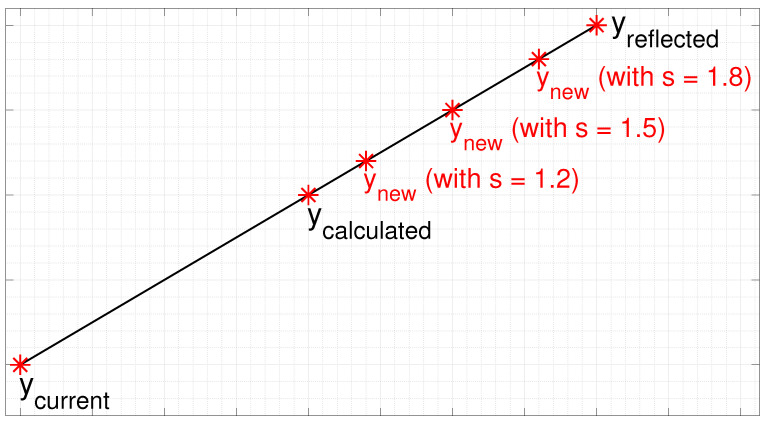
Example of $y_{\mathrm{new}}$ obtained using s=1.2, 1.5 and 1.8.

Lee et al. [26] defined that 1<s<2. The authors noted experimentally that for s<1, the algorithm converges more slowly compared to the LBG algorithm and for 1<s<2, the algorithm converges faster. For s=1, the algorithm corresponds to the LBG algorithm. Figure 2 illustrates the example of $y_{\mathrm{new}}$ obtained using s=1.2, s=1.5 and s=1.8.

## 3. Initialization Strategies

The LBG algorithm starts with an initial solution (initial codebook) that is iteratively improved to satisfy the centroid and nearest neighbor conditions, as described in Section 2. Initialization is a step that directly influences the performance of QV, as the convergence speed and the quality of the codebook obtained depend on the initial codebook.

A widely used initialization alternative is random initialization, in which the initial set of codevectors is drawn randomly from the training set. Other initialization methods can lead to a better quality of the designed codebooks.

Let $Z=\{{\vec{z}}_{q},q=1,2,\ldots ,Q\}$ be the training set, in which $\vec{z_{q}}=\left\{z_{q1},z_{q2},\ldots ,z_{qK}\right\}$ and let $Y_{0}=\left\{{\vec{y}}_{n},n=1,2,\ldots ,N\right\}$ be the initial codebook with *N* codevectors, in which $\vec{y_{n}}=\left\{y_{n1},y_{n2},\ldots ,y_{nK}\right\}$. The following subsections present several strategies for designing the initial codebook $Y_{0}$.

### 3.1. Hadamard

Hadamard initialization, introduced by Chen and Li in [9], is a technique for generating initial codevectors for VQ in the Hadamard domain. In the method, one initially calculates the Hadamard transform of the training vectors and orders the transformed vectors according to the value of their first components. Then, the sorted transformed vectors are partitioned into *N* groups and the inverse transform of the mid vector of each group is used as an initial codevector. The initial codebook is then composed of the *N* vectors obtained so far.

The detailed steps of the Hadamard technique are given below:

- Step 1: let $H_{K\times K}$ be the Hadamard matrix of dimension K×K. Calculate the transform of each input vector ${\vec{z}}_{q}$ as:
  (8) $$h\left({\vec{z}}_{q}\right)={\vec{z}}_{q}\cdot H_{K\times K},$$
  in which the Hadamard matrix is a square matrix with a dimension that is a power of 2, defined as follows:
  (9) $$H_{2^{t}}=\frac{1}{\sqrt{2^{t}}}\left[\begin{array}{cc} H_{2^{t-1}} & H_{2^{t}-1}\\ H_{2^{t-1}} & -H_{2^{t-1}} \end{array}\right],$$
  in which *t* is a non-negative integer and $H_{1}=\left(1\right)$. For example:
  (10) $$H_{4}=\frac{1}{\sqrt{4}}\left[\begin{array}{cccc} 1 & 1 & 1 & 1\\ 1 & -1 & 1 & -1\\ 1 & 1 & -1 & -1\\ 1 & -1 & -1 & 1 \end{array}\right].$$
- Step 2: sort the transformed training vectors in ascending order according to their first component.
- Step 3: divide the ordered vectors into *N* groups of equal size.
- Step 4: finally, select the mid vector of each ordered group, then $Y_{0}$ will be composed by the corresponding inverse Hadamard transform of the *N* selected mid vectors.

### 3.2. Group Strategy

In the Group Strategy initialization technique proposed by Ma et al. in [10], the authors use the variance and mean of the training set vectors to select the initial codebook for VQ.

The mean $\left(m_{{\vec{z}}_{q}}\right)$ and variance $\left(v_{{\vec{z}}_{q}}\right)$ of the vector ${\vec{z}}_{q}$ of dimension *K* are respectively defined by

(11) $$m_{{\vec{z}}_{q}}=\frac{1}{K}\sum_{j=1}^{K}z_{qj}$$

and

(12) $$v_{{\vec{z}}_{q}}=\frac{1}{K}\sum_{j=1}^{K}{\left(z_{qj}-m_{{\vec{z}}_{q}}\right)}^{2},$$

in which $z_{qj}$ is the *j*-th component of the vector ${\vec{z}}_{q}$.

The procedure for generating the initial codebook involves the following steps:

- Step 1: calculate the variance of all training vectors ${\vec{z}}_{q}$ according to Equation (12).
- Step 2: sort the training vectors in order of increasing variance.
- Step 3: divide the ordered vectors into 3 groups A, B and C, in the proportion 17:2:1, that is, ≈84% belong to group A, ≈10% to group B and the remainder (≈6%) to group C.
- Step 4: for Groups A and B, do:
  – Calculate the mean of each vector belonging to the group according to Equation (11);
  – Sort the vectors in ascending order according to the mean value;
  – Divide groups A and B into $\frac{N}{2}$ and $\frac{N}{4}$ subgroups, respectively;
  – Select the intermediate vector of each subgroup A and B as the codevector.
- Randomly select $\frac{N}{4}$ vectors from Group C.

### 3.3. Subtractive Clustering

The subtractive clustering technique, proposed by Mirzaei et al. in [11], benefits from the idea of subtractive clustering and density, so that when choosing a codevector for the initial codebook, the probability of choosing training vectors located nearby decreases.

The subtractive grouping technique can be stated as follows:

- Step 1: set the iteration counter i=1 and the maximum iteration counter *N*. Calculate the density of each vector $\vec{z_{q}}$ as follows:
  (13) $$D_{{\vec{z}}_{q}}=\sum_{j=1}^{Q}\exp\left(-\frac{∥{\vec{z}}_{q}-{\vec{z}}_{j}{∥}^{2}}{{\left(\frac{r_{a}}{2}\right)}^{2}}\right),$$
  in which $r_{a}$ is a positive constant that represents the radius of the neighborhood and ${\vec{z}}_{j}$ is a vector from the training set.
- Step 2: select the vector ${\vec{z}}_{q}$ that has the highest density $D_{\mathrm{MAX}}$ as the *i*-th codevector ${\vec{y}}_{i}$.
- Step 3: update the densities value of each vector $\vec{z_{q}}$ as follows:
  (14) $$D_{{\vec{z}}_{q}}=D_{{\vec{z}}_{q}}-D_{\mathrm{MAX}}\cdot \exp\left(-\frac{∥{\vec{z}}_{q}-{\vec{y}}_{i}{∥}^{2}}{{\left(\frac{r_{b}}{2}\right)}^{2}}\right),$$
  in which $r_{b}$ is a positive constant that defines a lower density neighborhood and ${\vec{y}}_{i}$ is the vector with the highest density $D_{\mathrm{MAX}}$.
- Step 4: if i=N, the method stops. Otherwise, do i=i+1 and return to Step 2.

### 3.4. KATSA

The central idea of the algorithm proposed by Katsavounidis, Kuo and Zhang [12] is based on the premise that the training vectors that are furthest from the others probably belong to different clusters. With this premise, the essence of the algorithm is to use the furthest training vector from the current codevectors as a new codevector. The algorithm can be described as follows:

- Step 1: calculate the norm of all training vectors and select the vector with the highest norm as the first codevector;
- Step 2: for each remaining training vector, find the closest codevector according to its Euclidean distance. The training vector that has the greatest distance in relation to its respective closest codevector is chosen to compose the codebook. This step is repeated until the codebook has the desired number of vectors.

### 3.5. MEIM

The initialization algorithm called Maximum Entropy Initialization Method (MEIM) was described by Nyeck and Tosser-Roussey in [13]. The method aims to ensure that the clusters of each initial codevector have similar sizes. Let $S=\{S_{i},i=1,2,\ldots ,N\}$ be the set of *N* clusters in which a training vector $\vec{z}$ belongs to the cluster $S_{i}$ if $N_{i}d\left(\vec{z},{\vec{y}}_{i}\right)<N_{j}d\left(\vec{z},{\vec{y}}_{j}\right)$ for all i≠j, where ${\vec{y}}_{i}$ and ${\vec{y}}_{j}$ are, respectively, the representation of $S_{i}$ and $S_{j}$, and $N_{i}$ is the size of $S_{i}$ and $N_{j}$ is the size of $S_{j}$. The algorithm is described as follows:

- Step 1: initialize the codebook $Y_{0}$ by randomly choosing *N* training vectors from *Z*;
- Step 2: initialize $N_{i}$ to 1 for all *i*;
- Step 3: for each training vector $\vec{z}$ evaluate if $N_{i}d\left(\vec{z},{\vec{y}}_{i}\right)<N_{j}d\left(\vec{z},{\vec{y}}_{j}\right),\forall i,j,$ where i≠j. If true, add $\vec{z}$ to $S_{i}$ and increment $N_{i}$;
- Step 4: calculate the set of centroids $C=\{\vec{c_{i}},i=1,2,\ldots ,N\}$ where
  (15) $${\vec{c}}_{i}=\frac{1}{N_{i}}\sum_{j=1}^{N_{i}}\left({\vec{z}}_{j}\in S_{i}\right);$$
- Step 5: take the final codebook as $Y=\{{\vec{y}}_{i},i=1,2,\ldots ,N\}$ where ${\vec{y}}_{i}\in S_{i}$ and ${\vec{y}}_{i}$ is the most similar codevector to ${\vec{c}}_{i}$.

### 3.6. DSICS

The algorithm called Double Sorting-Based Initial Codewords Selection (DSICS) was proposed by Hu et al. in [14]. The algorithm can be described as follows:

- Step 1: create a copy of the list of training vectors and name it $D_{1}$;
- Step 2: for each training vector in $D_{1}$, find the component with the lowest value and subtract this value from each component in its respective vector;
- Step 3: calculate the Euclidean distance of each vector in $D_{1}$ to the origin and sort $D_{1}$ in terms of the calculated distances;
- Step 4: make a new copy of the list of training vectors and name it $D_{2}$;
- Step 5: for each training vector in $D_{2}$, calculate the sum of its components and sort $D_{2}$ in terms of these calculations;
- Step 6: evenly divide $D_{1}$ and $D_{2}$ into *N* subsets, where *N* is the desired number of codevectors;
- Step 7: for each pair of corresponding sets, $d_{1i}$ and $d_{2i}$, if $d_{1i}\cap d_{2i}\neq \emptyset$, then select the median of $d_{1i}\cap d_{2i}$ as a codevector. Otherwise, select the median of $d_{1i}$ as a codevector.

## 4. Swarm Techniques Applied to VQ

Swarm algorithms are metaheuristic techniques inspired by nature, such as the behavior of collective beings such as bees [27], ants [28] and birds [29]. Swarm techniques have been applied to VQ codebook design [30,31,32].

The following subsections present three codebook design techniques based on bioinspired algorithms.

### 4.1. Modified FA-LBG Algorithm

Severo et al. proposed in [7] the M-FA-LBG (Modified Firelfly Algorithm—Linde–Buzo–Gray), developed from modifications made to the FA-LBG algorithm proposed by Horng in [33].

M-FA-LBG considers that a swarm has *P* fireflies, with each firefly representing a codebook $W^{\left(a\right)}$, with 1≤a≤P, of size *N* and dimension *K*, that is $W^{\left(a\right)}=\{{\vec{w}}_{i}^{\left(a\right)},$ i=1,2,…,N} where ${\vec{w}}_{i}^{\left(a\right)}$ represents the *i*-th codevector of the *a*-th codebook of dimension *K*, with ${\vec{w}}_{i}^{\left(a\right)}=\left\{w_{ij}^{\left(a\right)},\;j=1,2,\ldots ,K\right\}$, where $w_{ij}^{\left(a\right)}$ represents the *j*-th component of ${\vec{w}}_{i}^{\left(a\right)}$.

The objective of MA-FA-LBG is to find a codebook $W^{\left(a\right)}$ that maximizes the fitness function $f\left(W^{\left(a\right)}\right)$ for the input vectors:

(16) $$f\left(W^{\left(a\right)}\right)=\frac{1}{D\left(W^{\left(a\right)}\right)},$$

in which $D\left(W^{\left(a\right)}\right)$ is the distortion for the *a*-th codebook $W^{\left(a\right)}$, according to Equation (3).

In the FA-LBG, the codebook is iteratively updated through the repositioning of fireflies. Each firefly $W^{\left(a\right)}$ is attracted to a firefly $W^{\left(b\right)}$ with greater fitness and its movement is defined by

(17) $${\vec{w}}_{i}^{{\left(a\right)}^{t+1}}={\vec{w}}_{i}^{{\left(a\right)}^{t}}+\beta \cdot \left({\vec{w}}_{i}^{{\left(a\right)}^{t}}-{\vec{w}}_{i}^{{\left(b\right)}^{t}}\right)+\alpha \cdot \left(\vec{r}-{\vec{r}}_{\mathrm{aux}}\right),$$

in which α is the parameter that regulates the insertion of randomness in the path traversed by the firefly, $\vec{r}$ is a vector whose components are equal and correspond to a random number obtained from a uniform distribution between 0 and 1, ${\vec{r}}_{\mathrm{aux}}$ is a vector whose components are equal to 0.5 and β is the firefly’s attractiveness factor, defined by

(18) $$\beta =\beta_{0}\cdot \exp\left(-\gamma \cdot d^{2}\left({\vec{w}}_{i}^{{\left(a\right)}^{t}},{\vec{w}}_{i}^{{\left(b\right)}^{t}}\right)\right),$$

in which γ corresponds to the absorption coefficient of light through a medium, $d\left({\vec{w}}_{i}^{{\left(a\right)}^{t}},{\vec{w}}_{i}^{{\left(b\right)}^{t}}\right)$ is the Euclidean distance between any two fireflies and $\beta_{0}$ is the attractiveness of a firefly for $d\left({\vec{w}}_{i}^{{\left(a\right)}^{t}},{\vec{w}}_{i}^{{\left(b\right)}^{t}}\right)=0$. If there is no firefly with higher brightness, $W^{\left(a\right)}$ will move randomly, as follows

(19) $${\vec{w}}_{i}^{{\left(a\right)}^{t+1}}={\vec{w}}_{i}^{{\left(a\right)}^{t}}+\alpha \cdot \left(\vec{r}-{\vec{r}}_{\mathrm{aux}}\right).$$

M-FA-LBG proposes to precede the repositioning of fireflies with the calculation of the centroids of each Voronoi region. In this way, the codebook is updated by calculating the new centroids of the Voronoi regions, according to Equation (5) and then updating the position of the fireflies, according to Equations (17) and (19). The objective of introducing the centroid calculation in the codebook update step in M-FA-LBG is to allow a greater influence of the training set on the codebook design, aiming to minimize the distortion introduced when representing the training vectors by the corresponding code vectors.

The algorithm can be summarized in the following steps:

- Step 1 (initialization and parameter setting): let *Z* be training set. Divide the training set into *Q* vectors of *K* dimension, that is, $Z=\{{\vec{z}}_{q},q=1,\ldots ,Q\}$, in which $\vec{z_{q}}=\left\{z_{q1},z_{q2},\ldots ,z_{qK}\right\}$. Initialize the algorithm with *P* fireflies—each firefly corresponds to an initial random codebook, obtained from the training set. Set the parameters α, $\beta_{0}$ and γ used in the movement of the firefly and the distortion threshold ϵ that will be used as a stopping criterion.
- Step 2 (training set partitioning and search for the firefly with the greatest fitness): for each firefly $W^{\left(a\right)}$, do:
  – Allocate the *Q* training vectors in the *N* Voronoi regions according to Equation (2);
  – Calculate the centroid of each of the *N* Voronoi regions according to Equation (5);
  – Calculate the distortion according to Equation (3);
  – Search the codebook (firefly) with the highest fitness value according to Equation (16).
- Step 3 (position update of fireflies): update the positioning of each firefly $W^{\left(a\right)}$, with a=1,2,…,N, except the one with greatest brightness, according to Equation (17). If $W^{\left(a\right)}$ is the brightest firefly, update its position according to Equation (19).
- Step 4 (stop criterion): if the stopping criterion is satisfied according to Equation (6), the algorithm is finished with the output being the highest brightness firefly. Otherwise, return to Step 2.

### 4.2. Modified PSO-LBG Algorithm

The M-PSO-LBG (Modified Particle Swarm Optimization—Linde–Buzo–Gray), proposed by Severo et al. in [6], was derived from changes made to the PSO-LBG [34] allowing greater influence of the training set in codebook design. The changes concern the way the algorithm is initialized, the codebook training stage, that is, the codevectors update stage, and the definition of the algorithm’s stopping criterion.

In M-PSO-LBG, each particle, which correspond to a possible solution to the problem, corresponds to a codebook of size *N* and dimension *K*, that is $U^{\left(a\right)}=\left\{{\vec{u}}_{i}^{\left(a\right)},i=1,2,\ldots ,N\right\}$, with 1≤a≤P, where ${\vec{u}}_{i}^{\left(a\right)}$ represents the *i*-th codevector of the *a*-th codebook. The fitness function, which assesses each solution, is defined as

(20) $$f\left(U^{\left(a\right)}\right)=\frac{1}{D\left(U^{\left(a\right)}\right)},$$

where $D\left(U^{\left(a\right)}\right)$ is the distortion for the *a*-th codebook $U^{\left(a\right)}$, according to Equation (3).

In PSO, particles move in the search space based on their individual experience and the collective experience of the swarm [29]. The best position of particle *a*, $pbest^{\left(a\right)}$, represents the individual experience of each particle *a* and defines the best position (personal best) known to that particle. The position gbest represents the collective experience of the swarm and defines the best position (global best) known to the swarm as a whole.

In M-PSO-LBG, $pbest^{{\left(a\right)}^{t}}$ and $gbest^{t}$ must be updated as follows:

(21) $$pbest^{{\left(a\right)}^{t}}=\left\{\begin{array}{cc} U^{(a)t}, & \mathrm{if}\;f{\left(U^{\left(a\right)}\right)}^{t}>f{\left(U^{\left(a\right)}\right)}^{t-1}\\ pbest^{{\left(a\right)}^{t-1}}, & \mathrm{otherwise}, \end{array}\right.$$

and

(22) $$gbest^{t}=\left\{\begin{array}{cc} pbest_{\max}^{t}, & \mathrm{if}\;pbest_{\max}^{t}>gbest^{t-1}\\ gbest^{t-1}, & \mathrm{otherwise}, \end{array}\right.$$

in which $pbest^{{\left(a\right)}^{t}}=\left\{pbest_{i}^{{\left(a\right)}^{t}},\;i=1,2,\ldots ,N\right\}$, where $pbest_{i}^{{\left(a\right)}^{t}}$ represents the *i*-th pbest of the *a*-th codebook in iteration *t*, $gbest^{t}=\left\{gbest_{i},\;i=1,2,\ldots ,N\right\}$, where $gbest_{i}$ represents the *i*-th gbest in iteration *t*, $pbest_{\max}^{t}$ is the largest pbest value obtained among all particles in iteration *t*, at each iteration of the algorithm.

Each particle has a position vector and a velocity vector. The position vector stores the position of the particle in the search space. The velocity vector is responsible for indicating the direction in which changes in the position of each particle occur. In M-PSO-LBG, the position vector of each particle corresponds to the codevector of each codebook. The velocity vector ${\vec{v_{i}}}^{{\left(a\right)}^{t+1}}$ at iteration t+1 is calculated for each particle according to

(23) $${\vec{v_{i}}}^{{\left(a\right)}^{t+1}}=\omega {\vec{v_{i}}}^{{\left(a\right)}^{t}}+c_{1}r_{1}^{t}\vec{A}+c_{2}r_{2}^{t}\vec{B},$$

with

$$\vec{A}=pbest_{i}^{{\left(a\right)}^{t}}-{\vec{u}}_{i}^{{\left(a\right)}^{t}}\;\mathrm{and}\;\vec{B}=gbest_{i}^{t}-{\vec{u}}_{i}^{{\left(a\right)}^{t}},$$

in which ω is the inertia factor, $c_{1}$ is the particle’s cognitive acceleration constant, $c_{2}$ is the social acceleration constant and $r_{1}$ and $r_{2}$ are random coefficients that range from 0 to 1. After updating the velocity, the new position of the particle is calculated as

(24) $${\vec{u}}_{i}^{{\left(a\right)}^{t+1}}={\vec{u}}_{i}^{{\left(a\right)}^{t}}+{\vec{v_{i}}}^{{\left(a\right)}^{t+1}}.$$

The objective of M-PSO-LBG is to find a codebook that maximises the fitness function for the input vectors. With the aim of allowing a greater influence of the training set on the codebook design, M-PSO-LBG proposes to precede the repositioning of the particles with the calculation of the centroids of each Voronoi region, according to Equation (5). Therefore, updating each particle in M-PSO-LBG occurs in two steps:

1. First, with the update of the new centroids of the Voronoi regions of each of the *P* particles;
2. And then, with the updating of the velocity and position vectors of each of the particles.

The M-PSO-LBG can be summarised in the following steps:

- Step 1 (initialization and parameter setting): let *Z* be the training set. Divide the training set into *Q* vectors of *K* dimension, that is, $Z=\{{\vec{z}}_{q},q=1,2,\ldots ,Q\}$, in which $\vec{z_{q}}=\left\{z_{q1},z_{q2},\ldots ,z_{qK}\right\}$. Initialize the algorithm with *P* particles—each particle corresponds to an initial random codebook, obtained from the training set. Set the parameters used in calculating the velocity vector and the distortion threshold ϵ that will be used as a stopping criterion.
- Step 2 (training set partitioning and calculation of the fitness of each particle and updating pbest): for each particle, do:
  – Allocate the *Q* training vectors in the *N* Voronoi regions according to Equation (2);
  – Calculate the centroid of each of the *N* Voronoi regions according to Equation (5);
  – Calculate the distortion according to Equation (3);
  – Calculate the fitness function according to Equation (20);
  – Update position pbest according to Equation (21).
- Step 3 (gbest update): search the particle with the highest fitness and update the position gbest according to Equation (22).
- Step 4 (update particle velocity and position): the velocity of each particle is updated in two steps:
  – First, the velocity ${\vec{v_{i}}}^{{\left(a\right)}^{t}}$ at iteration *t* is calculated for each particle according to
    (25) $${\vec{v_{i}}}^{{\left(a\right)}^{t}}={\vec{u}}_{i}^{{\left(a\right)}^{t}}-{\vec{u}}_{i}^{{\left(a\right)}^{t-1}},$$
    which corresponds to the difference between the particle update before and after the centroid calculation. This is done to guarantee the convergence of the M-PSO-LBG, so that the particle follows a convergence trajectory based on the characteristics of the LBG algorithm, since the particle positioning update is preceded by its update based on the centroid calculation.
  – In the second step, the velocity vector is updated according to Equation (23). And then, the new position of the particle is calculated according to Equation (24).
- Step 5 (stop criterion): if the stopping criterion is satisfied according to Equation (6), the algorithm is finished, with the output being the gbest. Otherwise, return to Step 2.

### 4.3. Modified FSS-LBG Algorithm

The M-FSS-LBG (Modified Fish School Search—Linde–Buzo–Gray) was proposed by Fonseca, Ferreira and Madeiro in [8] which is a vector quantization codebook design method based on the Fish School Search (FSS) swarm algorithm [35]. The M-FSS-LBG algorithm has as a metaphor the behavior of a school of fish in search for food where each fish represents a different codebook. In addition to the conventional steps of the LBG algorithm, in M-FSS-LBG some movement rules are applied to move the fish in the search space (individual movement, instinctive movement and volitive movement). Furthermore, a feeding operator is used to evaluate the success of a new movement and a breeding operator is applied to increase the exploration capacity of the algorithm. The fitness function of a given fish in the M-FSS-LBG algorithm is the inverse of its general distortion.

In the Individual Movement, each fish moves randomly in its neighborhood. For a given position ${\vec{p}}_{i}$, this movement is given by

(26) $${\vec{p}}_{i}^{t}={\vec{p}}_{i}^{t-1}+\left[-1,1\right]\alpha_{\mathrm{ind}},$$

where ${\vec{p}}_{i}^{t}$ is the position of the *i*th fish in the current iteration and ${\vec{p}}_{i}^{t-1}$ is the position of the *i*th fish in the previous iteration, $\left[-1,1\right]$ is a pseudo-random number generated by a uniform distribution in the range $\left[-1,1\right]$ and $\alpha_{\mathrm{ind}}$ is a parameter that controls the individual movement rate. After the movement, it is evaluated whether the new position is better than the previous one in terms of the fitness function. If it is not better, the fish returns to its previous position.

With each successful Individual Movement the weight of the respective fish increases according to a Feeding Operator. If the Individual Movement of the fish is unsuccessful, its weight is reduced. The weight of each fish is determined by

(27) $$W_{i}^{t}=W_{i}^{t-1}+\frac{\Delta f_{i}}{\max\left(\Delta f\right)},$$

where $\Delta f_{i}$ is the difference between the current and previous fitness of the *i*th fish and $\max\left(\Delta f\right)$ is the maximum $\Delta f_{i}$ among all fish. To control the growth of fish weight, a parameter is used to limit the maximum weight.

Every successful Individual Movement will also influence the fish to make an Instinctive Movement in a resulting collective direction $\vec{I}$, given by

(28) $$\vec{I}=\frac{\sum_{i=1}^{P}\Delta {\vec{p}}_{i}\Delta f_{i}}{\sum_{i=1}^{P}\Delta f_{i}},$$

where *P* is the number of fish in the school and $\Delta {\vec{p}}_{i}$ is the difference between the current and previous positions of the *i*th fish. Each fish moves towards that direction, that is,

(29) $${\vec{p}}_{i}^{t}={\vec{p}}_{i}^{t}+\vec{I}.$$

The Volitive Movement aims to control the exploration and exploitation of the school. If the total weight of the school is greater in relation to the total weight in the previous iteration, then the entire school must move in the direction of its barycenter $\vec{B}$ (favoring exploitation). Otherwise, they move in the opposite direction (favoring exploration). The barycenter is given by

(30) $$\vec{B}=\frac{\sum_{i=1}^{P}{\vec{p}}_{i}W_{i}}{\sum_{i=1}^{P}W_{i}}.$$

According to the calculated barycenter, the school moves as

(31) $${\vec{p}}_{i}^{t}=\left\{\begin{array}{cc} {\vec{p}}_{i}^{t}-\alpha_{\mathrm{vol}}\left[0,1\right]\frac{{\vec{p}}_{i}^{t}-\vec{B}}{d\left({\vec{p}}_{i}^{t},\vec{B}\right)}, & \mathrm{if}\;\Delta W>0\\ {\vec{p}}_{i}^{t}+\alpha_{\mathrm{vol}}\left[0,1\right]\frac{{\vec{p}}_{i}^{t}-\vec{B}}{d\left({\vec{p}}_{i}^{t},\vec{B}\right)}, & \mathrm{otherwise}, \end{array}\right.$$

where $\alpha_{\mathrm{vol}}$ is the parameter that controls the rate of the Volitive Movement and ΔW is the difference between the total weight in the current and previous iterations.

At last, the Breeding Operator is applied to provide greater variety among fish and favor the exploration of the search space. This operator is given as follows:

- Step 1: the current worst fish is replaced by breeding itself with the best fish in the current school;
- Step 2: two random fish are chosen from the remaining P−2 fish;
  – Step 2.1: the first random fish is replaced by breeding itself with the second random fish;
  – Step 2.2: the second random fish is replaced by breeding itself with the best fish in the current school.

The breeding itself is given by calculating the arithmetic average of the weights and positions of two fish, to define the weight and position of the new fish.

The following steps summarize the M-FSS-LBG algorithm:

- Step 1: initialize *P* fish with *N* codevectors each;
- Step 2: for each fish:
  – Step 2.1: for each training vector search the nearest neighbor in the respective fish;
  – Step 2.2: assign the training vector to the Voronoi region according to Step 2.1;
- Step 3: evaluate the LBG stopping criterion considering the fish whose fitness function is the highest;
  – Step 3.1: if the stopping criterion is satisfied, go to Step 11;
  – Step 3.2: otherwise, continue;
- Step 4: for each fish apply the Individual Movement;
- Step 5: for each fish apply the Feeding Operator;
- Step 6: for each fish apply the Instinctive Movement;
- Step 7: for each fish apply the Volitive Movement;
- Step 8: for each fish apply the Breeding Operator;
- Step 9: for each fish:
  – Step 9.1: for each training vector search the nearest neighbor in the respective fish;
  – Step 9.2: assign the training vector to the Voronoi region according to Step 9.1;
- Step 10: for each fish update the centroids as in Equation (5) and go to Step 2;
- Step 11: return the fish whose fitness function is the highest.

## 5. Metodology

This section presents the methodology used in the experimental setup.

The codebooks were designed with dimension K=16, that is, image blocks of 4×4 pixels, and sizes *N* = 32, 64, 128, 256 and 512.

The following notation is used for the considered algorithms: modified FA-LBG algorithm (M-FA-LBG), modified PSO-LBG algorithm (M-PSO-LBG) and modified FSS-LBG algorithm (M-FSS-LBG); the algorithms M-FA-LBGa, M-PSO-LBGa and M-FSS-LBGa correspond to an accelerated version, respectively, of M-FA-LBG, M-PSO-LBG and M-FSS-LBG, obtained using the technique proposed by Lee et al. [26] (see Section 2.2).

The threshold used as a stopping criterion for the algorithms was ϵ=0.001. The scaling factors of the LBGa algorithm used in M-FF-LBGa, M-PSO-LBGa and M-FSS-LBGa were, respectively, 1.7, 1.4 and 1.7. The value of this parameter was obtained from a previous analysis carried out by varying the scale factor between 1.1 and 1.9. The average values of peak signal-to-noise ratio (PSNR) were computed. The scale factors which resulted in the largest PSNR values were chosen.

The parameters used in each algorithm are presented in Table 1. The values presented in Table 1 were obtained by preliminary simulations carried out with thirty executions for each algorithm in different sets of parameters considering each initialization strategy. Average PSNR values were computed for each set of parameters. The parameters which resulted in the largest PSNR values were chosen.

**Figure 3:**
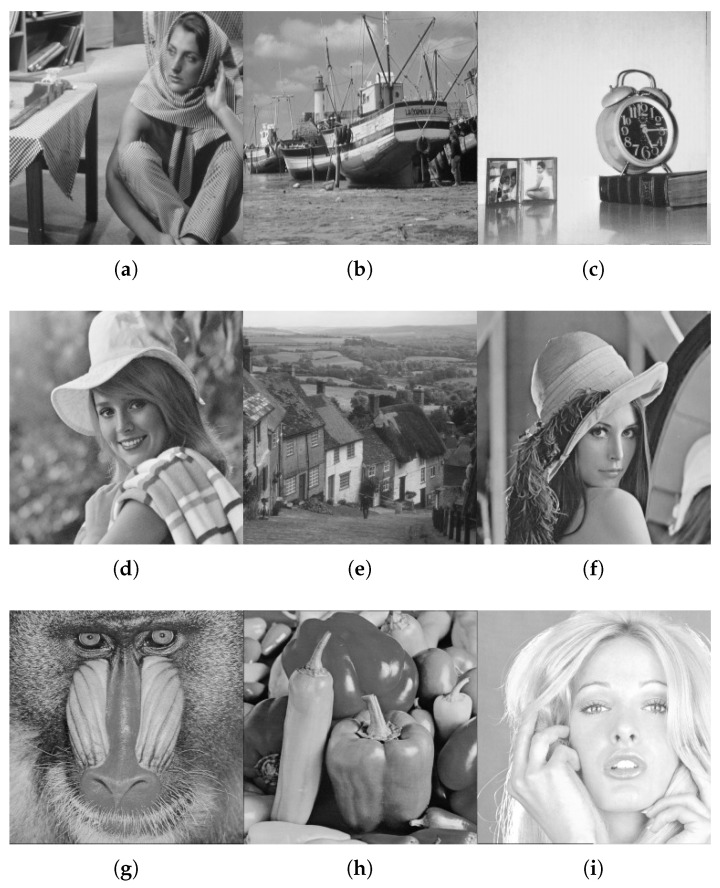
Images (**a**) Barbara, (**b**) Boat, (**c**) Clock, (**d**) Elaine, (**e**) Goldhill, (**f**) Lena, (**g**) Mandrill, (**h**) Peppers, (**i**) Tiffany used in the simulations.

The images Barbara, Boat, Clock, Elaine, Goldhill, Lena, Mandrill, Peppers and Tiffany were used as training sets. The images are 256×256 pixels, portable gray map (PGM), 8.0 bits per pixel (bpp). Figure 3 presents the images used in the simulations.

For each swarm technique used, ten particles were used, therefore, in each execution of the technique, ten codebooks are initialized, where each codebook corresponds to a particle. This value was chosen based on a previous analysis of the performance of the algorithms for population sizes P=10,20,30,50 and 100. As *P* increases, the number of mathematical operations performed by the algorithms increases, consequently the execution time of the algorithms increases. It was observed for the *p* values analyzed that the PSNR varies little with the increase in population size. Therefore, taking into account the computational cost with increasing population size and the performance in terms of PSNR obtained for each size, it was decided to use P=10.

Thirty executions of the algorithms were performed and the results were obtained in terms of the arithmetic mean at the end of the executions. Therefore, for each image thirty codebooks were designed, for each codebook of size *N*, with different initialization approaches.

The initialization techniques used were Random Selection, Hadamard Initialization, Group strategy, Subtractive Clustering, DSICS, MEIM and KATSA. Initializations were performed considering groups of techniques. Table 2 presents the composition of the initialization techniques used. Each letter used in the acronym represents an initialization. In “Random” initialization, the 10 codebooks are initialized randomly, while in the remaining initializations, combinations of the initialization techniques presented in Section 3 are used and the rest of the initial codebooks are randomly obtained from the training set. For example, in the SH strategy, one codebook is initialized by the Subtractive Clustering technique (S), one by the Hadamard technique (H) and the remaining eight are initialized randomly.

The quality of the designed codebooks was evaluated by the average PSNR given by

(32) $$\mathrm{PSNR}(\mathrm{dB})=10\log_{10}\left[\frac{L^{2}}{\mathrm{MSE}}\right],$$

in which *L* is the peak amplitude value of the input image. In the case of 8 bpp original image (256 gray levels), one has L=255.

Let *I* and $I^{\prime }$ be two digital images of $T_{1}\times T_{2}$ pixels, where *I* is the original image and $I^{\prime }$ the reconstructed image (VQ result). The MSE (mean squared error) between images *I* and $I^{\prime }$ is defined as

(33) $$\mathrm{MSE}=\frac{1}{T_{1}\times T_{2}}\sum_{r=0}^{T_{1}-1}\sum_{c=0}^{T_{2}-1}{\left[I\left(r,c\right)-I^{\prime }\left(r,c\right)\right]}^{2},$$

in which I(r,c) and $I^{\prime }\left(r,c\right)$ represent respectively the pixel values of the original and reconstructed images in *r*-th row and *c*-th column, with $T_{1}$ representing the number of rows and $T_{2}$ the number of columns of an image.

In addition to evaluating the quality of the reconstructed images, the algorithms were evaluated in terms of convergence speed, using the average number of iterations.

The statistical tools Friedman test and Nemenyi test were used to compare the average results of PSNR and number of iterations obtained.

The Friedman test [36] is a statistical test that analyzes the existence of a statistically significant difference between the $n_{\mathrm{models}}$ models in the $n_{\mathrm{data}\mathrm{sets}}$ data sets. The objective of the Friedman test is to inform whether there is a statistical difference between the compared methods [37]. In this work, the Friedman test was applied with $n_{\mathrm{models}}=10$ codebook initialization strategies, $n_{\mathrm{data}\mathrm{sets}}=30$ codebook sets and a significance level of 5%. If the *p*-value found is less than the desired level of significance (0.05), then there is a significant difference between the performance of the initialization strategies used. In this scenario, the Friedman test is not able to indicate whether all strategies are different or whether only one of them performs significantly differently, while the others can be considered statistically equal. The Nemenyi test [38] is a post-test used to detect which differences between models are significant.

The Nemenyi test is used to compare models “one by one”, indicating where the significant differences are [37]. The difference in performance between two initialization techniques is statistically significant if their respective average ratings differ by at least one critical difference (CD) [37,39]:

(34) $$\mathrm{CD}=q_{\alpha }\cdot \sqrt{\frac{n_{\mathrm{models}}\cdot \left(n_{\mathrm{models}}+1\right)}{6\cdot n_{\mathrm{data}\mathrm{sets}}}},$$

where $q_{\alpha }$, for the significance level α, is obtained from the table of critical values for the Nemenyi test, available in [37].

**Figure 4:**
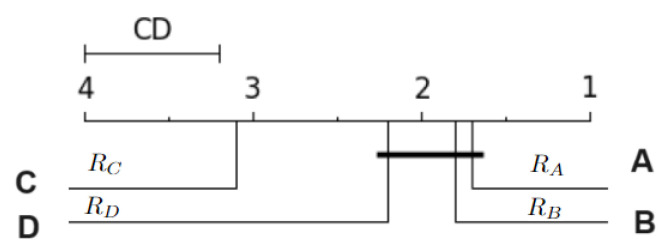
CD diagram example.

In this work, the results of the Nemenyi test were illustrated in the CD diagram [37]. Figure 4 shows an example of a CD diagram, with four hypothetical classifiers: A, B, C and D. The CD value is displayed as a line at the top of the diagram. The horizontal axis of the diagram shows the average ranks, ordered from right to left. The identification of the classifiers and their respective average ranks appear next to each vertical line, below the axis. The connected groups are not significantly different from each other, at the 0.05 significance level. For example, in Figure 4, classifier A has the best average rank, but is statistically equivalent to classifiers B and D.

## 6. Results

For each image and for each *N* (codebook size), ten different initialization strategies were evaluated with the algorithms M-PSO-LBG, M-PSO-LBGa, M-FSS-LBG, M-FSS-LBGa, M-FF-LBG and M-FF-LBGa.

Table 3 presents the average PSNR results in dB for the Clock image obtained with the M-PSO-LBG algorithm. Among the initialization strategies used, the one that presented the best performance, for codebook sizes smaller than or equal to 128, was the MeKt strategy. For N=256 and N=512, the DsKt and MeKt strategies presented the best results. For all strategies, it is observed that the superiority over random initialization, in terms of PSNR, of the Clock image, increases with *N*. In particular, for N=512, the average PSNR gain obtained by replacing random initialization with DsKt or MeKt is 4.43 dB.

**Figure 5:**
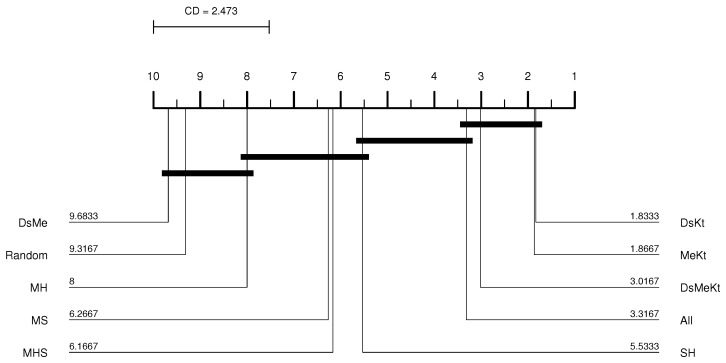
CD diagram of the Clock image considering the average PSNR (N=512 and M-PSO-LBG).

Performing the Friedman test with a significance of 5% (95% confidence) between the different initialization strategies, it was observed that with M-PSO-LBG, considering the average PSNR results, the *p*-value presented was always less than 0.05 as shown in Table 4. This means that there is a statistical difference between the compared initialization strategies. Once the statistical difference was evident, the Nemenyi test was applied. According to the Nemenyi test, two initialization strategies contain significant differences if their respective average rankings differ by at least one critical difference. The Nemenyi test was applied with CD=2.473 and $q_{0.05}=3.164$ (see Equation (34)). Figure 5 shows the resulting CD diagram of the Clock image with N=512 for the M-PSO-LBG algorithm, where the horizontal axis represents the average ranking of initialization strategies. The lines below the horizontal axis connect the strategies that do not contain a significant statistical difference with 95% confidence. The diagram shows that the DsKt and MeKt strategies, in this order, are better positioned in the ranking. Furthermore, it is noted that there is no statistical superiority between the strategies that contain the initialization Kt and they present superiority in relation to the other strategies.

Table 5 presents the average number of iterations for the Clock image obtained with the M-PSO-LBG algorithm. For *N* less than or equal to 128, any initialization strategy performs better than the random strategy. For N=256, the worst performance occurs for the MH strategy and the best performance occurs for the All strategy. For N=512, the best performance is from the DsMe strategy with 19.00 iterations on average. It is important to highlight that, for N=512, an average of 20.13 iterations were needed to obtain the average PSNR value of 31.20 dB for random initialization. Note that for the DsKt initialization (which presents the best result in terms of PSNR among the evaluated strategies), 34.70 average iterations were required to obtain an average PSNR of 35.63 dB. The “price” for increasing the average PSNR for N=512 obtained by replacing the random strategy with the DSKt strategy is maybe “paid” by the average number of iterations. However, this result can still be improved using the accelerated version of M-PSO-LBG (M-PSO-LBGa algorithm).

Table 6 presents the average PSNR results in dB for the Clock image obtained with the M-PSO-LBGa algorithm. For N=32, the DsKt initialization presents the best result. For N=64, N=256 and N=512, strategies that contain the Kt initialization show better results; a similar behavior is observed for N=128, where the DsMeKt strategy presents the highest average PSNR value (29.67 dB) and the other strategies that contain the Kt initialization present results very close to 29.67 dB. In general, in most cases, a lower average number of iterations is observed for M-PSO-LBGa compared to M-PSO-LBG. Note, comparing Table 7 and Table 5, that a smaller average number of iterations is required for the DsKt strategy with the M-PSO-LBGa algorithm compared to the M-PSO-LBG algorithm. For N=256 and N=512, the DskT strategy with M-PSO-LBGa presents an average number of iterations of 18.00 and 21.00, respectively. While for M-PSO-LBG the DskT strategy for N=256 and N=512 presents an average number of iterations of 51.53 and 34.70, respectively. This corresponds to a reduction in the average number of iterations of approximately 65% and 39%, respectively.

Table 8 and Table 9 present the average PSNR results of the Clock image obtained by the M-FSS-LBG and M-FSS-LBGa algorithms, respectively. For both the M-FSS-LBG algorithm and its accelerated version, the MS initialization strategy, for codebook sizes less than or equal to 256 presented the best performance in terms of average PSNR compared to the results obtained with the other initialization strategies. For N=512, the All strategy presented the best average PSNR result for both M-PSO-LBG and its accelerated version. In particular, for N=512, for both M-PSO-LBG and M-PSO-LBGa, the All strategy presented an average PSNR gain over random initialization greater than 3 dB.

Table 4 presents the Friedman test performed between the PSNR results obtained with the M-FSS-LBG and M-FSS-LBGa algorithms. For all codebook sizes *N*, *p*-value presented a result below 0.05, indicating the existence of a significant statistical difference.

**Figure 6:**
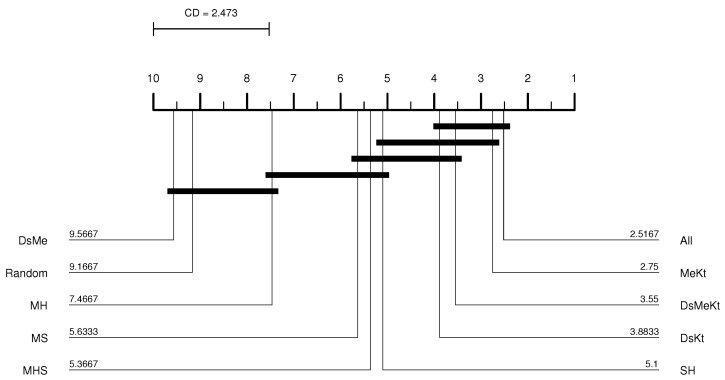
CD diagram of the Clock image considering the average PSNR (N=512 and M-FSS-LBG).

**Figure 7:**
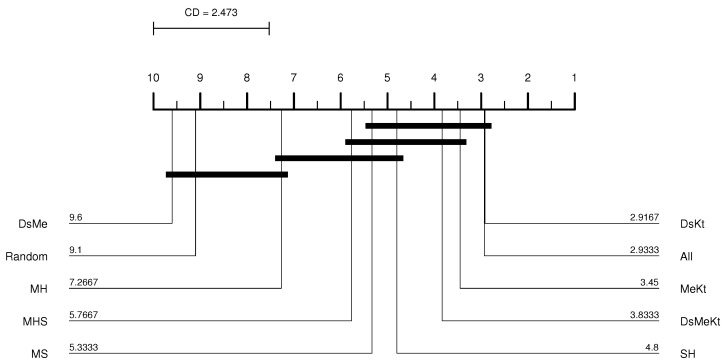
CD diagram of the Clock image considering the average PSNR (N=512 and M-FSS-LBGa).

From the CD diagram of the Clock image with N=512 (M-FSS-LBG algorithm), presented in Figure 6, it is possible to see that the All strategy was the best placed in the ranking and that it presents statistical superiority in relation to the SH, MHS, MS, MH, DsMe and random strategies. For the M-FSS-LBGa algorithm, the CD diagram, presented in Figure 7, shows that the DsKt and All strategies are positioned as first and second, respectively, in the ranks, and that they present statistical superiority in relation to the MHS, MH, DsMe and random strategies.

Regarding the average number of iterations, for N=256 and N=512, the M-FSS-LBG algorithm required a smaller number of average iterations compared to the M-FFS-LBGa algorithm, as can be seen in Table 10 and Table 11. For example, for M-FSS-LBG with N=256, the SH initialization strategy had the lowest average number of iterations with 17.10, while for M-FSS-LBGa, the SH initialization strategy had an average number of 21.87 iterations. For M-FSS-LBG with N=512, the MeKt initialization strategy had the lowest average number of iterations with 12.00, while for M-FSS-LBGa, the MeKt initialization strategy had an average number of 14.90 iterations. For N=64 and N=128, M-FSS-LBGa presented the best results in terms of average number of iterations with the All strategy. For N=32, M-FSS-LBG provided the best result with the All strategy, which presented an average number of iterations of 19.70.

**Figure 8:**
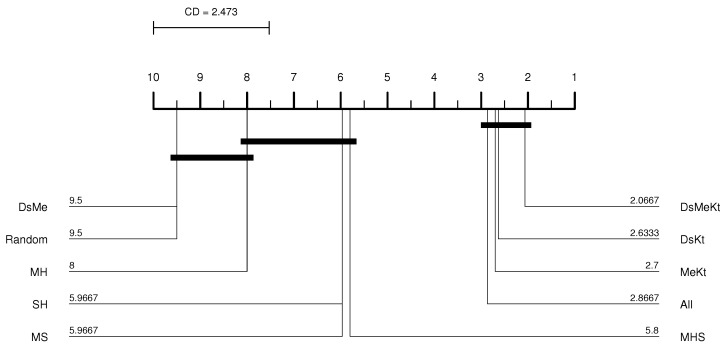
CD diagram of the Clock image considering the average PSNR (N=512 and M-FF-LBG).

For the Clock image, for the M-FF-LBG and M-FF-LBGa algorithms, only for N=32 and N=256 the initialization strategy that presented the best performance was the same, more specifically, the strategy DsMeKt for N=32 and the DsKt strategy for N=256, as can be seen in Table 12 and Table 13. The most significant gain over Random initialization is observed for N=512 (M-FF-LBG) with the DsMeKt initialization strategy, with a gain of 3.11 dB. It is important to highlight that, for N=512, for both M-FF-LBG and M-FF-LBGa, the strategies that use the Kt initialization present very close results, with values varying between 35.66 dB and 35.69 dB for the M-FF-LBG and between 35.49 dB and 35.50 dB for the M-FF-LBGa. The CD diagram of the results obtained with the M-FF-LBG algorithm for the Clock image with N=512, presented in Figure 8, indicates that the DsMeKt strategy was the best placed in the ranking and presents statistical superiority in relation to strategies that do not use the Katsa initialization.

Regarding the average number of iterations, comparing the Clock image results obtained with the M-FF-LBG and M-FF-LBGa algorithms, presented, respectively, in Table 14 and Table 15, it is observed that the M-FF-LBGa algorithm requires a lower average number of iterations. For example, for N=512, the M-FF-LBG algorithm with the MHS initialization strategy requires an average of 54.80 iterations, while the accelerated version of M-FF-LBG requires an average of 16.50 iterations. This corresponds to a reduction in the average number of iterations of approximately 70%.

The average PSNR results in dB of the Elaine image with M-PSO-LBG are presented in Table 3. For N≤128, the average PSNR results obtained with the initialization strategies were very close to the results obtained with Random initialization; the MS strategy presented the best result. For N=256, the MH initialization strategy presented the best performance compared to the results obtained with the other initialization strategies. For N=512, replacing the Random initialization strategy with MeKt initialization strategy leads to a PSNR gain of 1.32 dB.

For the Elaine image, for N≤256, the results obtained in terms of average PSNR by M-PSO-LBGa (Table 6) are similar to those of M-PSO-LBG. It is worth mentioning that, for Random initialization with N=512, using the M-PSO-LBGa in substitution to M-PSO-LBG, it was possible to achieve a PSNR gain of 1.01 dB.

Regarding the average number of iterations, comparing the Elaine image results obtained by the M-PSO-LBG and M-PSO-LBGa algorithms, presented, respectively, in Table 5 and Table 7, it is observed that M-PSO-LBGa presents better performance compared to the results obtained with M-PSO-LBG, except for the random and DsMe strategies with N=512. For example, for N=512, with the random initialization strategy, M-PSO-LBGa presented an average of 51.73 iterations and M-PSO-LBG presented an average of 19.00 iterations. As for the DsMe initialization strategy, for N=512, M-PSO-LBGa presented an average of 58.37 iterations and M-PSO-LBG presented an average of 22.93 iterations. This implies an increase of approximately 172% and 154% in the number of iterations, respectively, for the Random and DsME strategies, substituting M-PSO-LBG by M-PSO-LBGa.

Concerning the average PSNR results for the image Elaine with the M-FSS-LBG, presented in Table 8, the MH strategy presented the best results for N=32 and N=64. For N=128 and N=256, the MHS strategy presented the best result. For N=512, the average PSNR gain obtained by replacing Random initialization with DsKt is 0.57 dB. The average PSNR results of the Elaine image with the accelerated version of M-PSO-LBG are presented in Table 9. The results obtained by M-FSS-LBGa are quite similar to the ones of M-PSO-LBG. For example, for N=512 with the DsMeKt initialization strategy, M-FSS-LBGa obtained a PSNR of 34.02 dB and M-FSS-LBG obtained 34.00 dB.

Comparing the results of the average number of iterations for the Elaine image, for N=32, M-FSS-LBG (Table 10) presented better results than the ones of M-FSS-LBGa (Table 11) for most initialization strategies. For N=64, with M-FSS-LBG, the MS initialization strategy presented the best result with an average of 16.03 iterations. For M-FSS-LBGa the MHS strategy presented the best result with an average of 16.60 iterations. For N=128 and N=256, with M-FSS-LBG, the All initialization strategy presented the best result. With M-FSS-LBGa, for N=128 and N=256, the MHS strategy presented the best result. For N=512, with M-FSS-LBG, replacing the Random strategy with the MeKt strategy showed a reduction of 45% in the average number of iterations. With M-FSS-LBGa, replacing the Random strategy with the DsKt strategy, for N=512, showed a reduction of approximately 47% in the average number of iterations.

Table 12 and Table 13 present the average PSNR results with M-FF-LBG and M-FF-LBGa, respectively, for the Elaine image. For M-FF-LBG, the All strategy presented the best performance for N=256 and the strategies containing the Kt initialization presented better results for the codebook designed with N=512. For N=512, the Random initialization obtained a PSNR of 33.86 dB and the strategies that contain the Kt initialization obtained 34.22 dB. For the accelerated version of M-FF-LBG, with N=256, Random initialization presented the best result, while for N=512, the DsMe strategy presented the best result.

**Figure 9:**
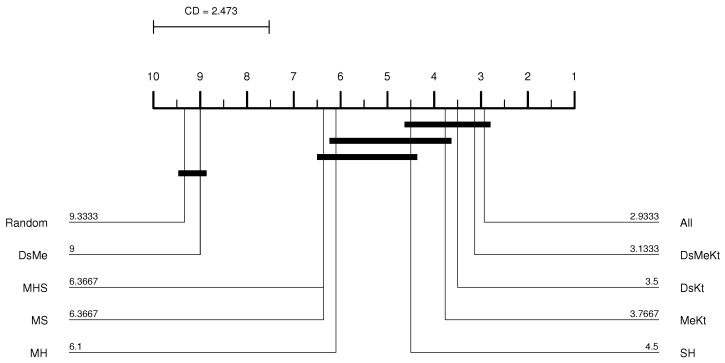
CD diagram of the Elaine image considering the average PSNR (N=512 and M-FF-LBG).

**Figure 10:**
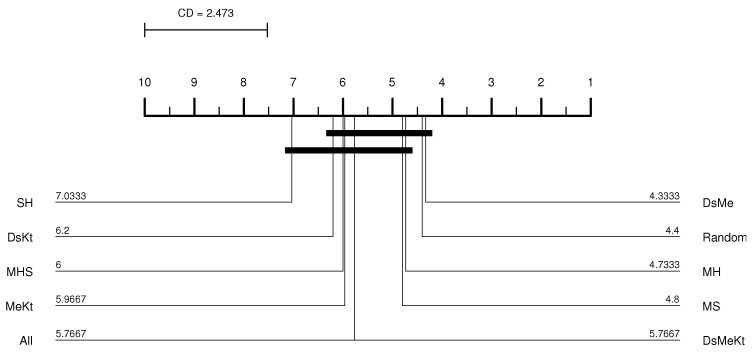
CD diagram of the Elaine image considering the average PSNR (N=512 and M-FF-LBGa).

It is observed that using the Friedman test performed between the PSNR results obtained with M-FF-LBG and M-FF-LBGa (Table 16), the *p*-value presented a result below 0.05 for all *N* codebook sizes, indicating the existence of a significant statistical difference. The Nemenyi test for M-FF-LBG presented in Figure 9 shows that the best average PSNR results for N=512 were obtained with the strategies that contain the Kt initialization and that there is no significant difference between these results and the results obtained with the SH strategy. For the accelerated version of M-FF-LBG, with N=512, the Nemenyi test presented in Figure 10 indicates that there is a significant difference in terms of average PSNR only between the SH strategy and the DsMe and Random strategies.

Table 14 and Table 15 present, for the Elaine image, the results of the average number of iterations for M-FF-LBG and M-FF-LBGa, respectively. For N≤256, the results obtained with M-FF-LBGa surpass the results obtained with M-FF-LBG. For example, the SH strategy for N=32 presented an average number of 39.00 iterations with M-FF-LBG, while M-FF-LBGa presented an average number of 28.23 iterations. For N=512, all initialization strategies, except the DsMe and Random strategies, presented better results with the replacement of M-FF-LBG by its accelerated version. For example, the DsKt strategy presented an average number of 36.73 iterations with M-FF-LBGa and an average number of 53.90 iterations with M-FF-LBG. This implies a reduction of approximately 32% in the average number of iterations.

Table 3 and Table 6 present the average PSNR results obtained with the Peppers image for M-PSO-LBG and M-PSO-LBGa, respectively. It is observed that for N≤128, the results obtained by M-PSO-LBG are similar to the ones of M-PSO-LBGa. For N≥256, the results obtained with M-PSO-LBG were better than the ones of M-PSO-LBGa. For example, for N=256 with the MH initialization strategy, the M-PSO-LBG presented an average PSNR of 30.60 dB and the M-PSO-LBGa presented an average PSNR of 30.43 dB. For N=512 with the DsKt initialization strategy, the M-PSO-LBG presented an average PSNR of 33.01 dB and the M-PSO-LBGa presented an average PSNR of 32.83 dB.

**Figure 11:**
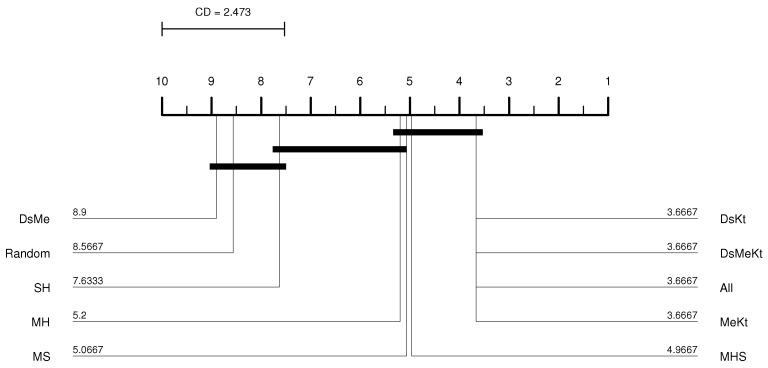
CD diagram of the Peppers image considering the average PSNR (N=512 and M-PSO-LBGa).

The results presented in Table 17 for the Friedman test with the accelerated version of M-PSO-LBG show that there is no significant difference in terms of average PSNR for N≤64. Meanwhile, for N≥128, the *p*-value was less than 0.05, indicating the existence of a statistically significant difference in terms of average PSNR. The CD diagram of the results obtained with the M-PSO-LBGa algorithm for the Peppers image, with N=512, presented in Figure 11, indicates that the strategies that contain the Kt initialization were the best placed in the ranking and present statistical superiority in relation to the Random, DsMe and SH strategies.

Regarding performance in terms of the average number of iterations, the results obtained with M-PSO-LBGa (Table 7) overperform the results obtained with M-PSO-LBG (Table 5) for N≤128. For N=256, the results obtained with M-PSO-LBGa outperform the results obtained with M-PSO-LBG, except for the DsKt initialization strategy. It is observed that for N=512, M-PSO-LBG overperforms M-PSO-LBGa only for the MH, MS and MHS strategies. For N=512, the M-PSO-LBG presented an average number of 49.20 iterations with the DsKt initialization strategy. When replacing M-PSO-LBG by M-PSO-LBGa, the average number of iterations, for N=512 with the DsKt initialization strategy, reduces to 22.00 iterations. This represents savings of more than 55%.

**Figure 12:**
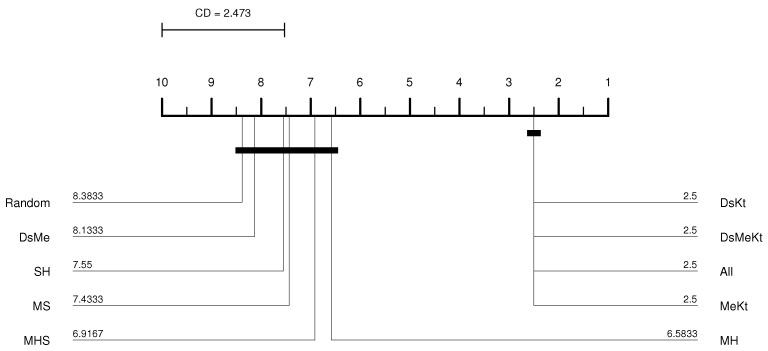
CD diagram of the Peppers image considering the average number of iterations (N=512 and M-PSO-LBGa).

Table 18 presents the Friedman test for the results of the average number of iterations obtained with M-PSO-LBGa. The results indicate the existence of significant difference in the codebooks with N≥64. For N=512, strategies that contain the Kt initialization present the best results. This can be seen in the CD diagram presented in Figure 12, which shows the best placed strategies in the ranking. Note that the strategies that contain the initialization Kt present statistical superiority in relation to the other strategies.

Table 8 and Table 9 present the average PSNR results obtained for the Peppers image with M-FSS-LBG and M-FSS-LBGa, respectively. For both M-FSS-LBG and M-FSS-LBGa, only for N≥128, there are significant differences in the results between the initialization strategies. This can be seen in Table 17, which presents the results of the Friedman test in terms of average PSNR for the M-FSS-LBG and its accelerated version, considering the Peppers. The *p*-value presented a result below 0.05 for N≥128, indicating the existence of significant statistical difference between the initialization strategies under consideration. For example, considering M-FSS-LBG, the All initialization strategy obtained an average PSNR gain over Random initialization of 0.29 dB for N=256 and an average PSNR gain of 0.60 dB for N=512. Considering M-FSS-LBGa, the MH initialization strategy obtained an average PSNR gain over Random initialization of 0.19 dB for N=256 and the All initialization strategy obtained an average PSNR gain over Random initialization of 0.73 dB for N=512.

Table 10 and Table 11 present the results of the average number of iterations for M-FSS-LBG and M-FSS-LBGa, respectively, with the Peppers image. It is observed that by replacing random initialization with an initialization strategy it is possible to obtain reductions in the average number of iterations in the range of 0.33 to 10.33 for M-FSS-LBG and in the range of 0.74 and 7.60 for M-FSS-LBGa. For example, for M-FSS-LBG and N=512, Random initialization obtained an average number of iterations of 37.60 and the All strategy obtained 27.27 iterations, that is, a difference of 10.33 iterations. For the accelerated version of M-FSS-LBG, for N=256, Random initialization obtained an average number of iterations of 33.70 and the All strategy obtained 26.10 iterations, that is, a difference of 7.60 iterations.

Table 12 and Table 13 present the average PSNR results obtained with M-FF-LBG and its accelerated version, respectively, considering the Peppers image. It is observed that for both M-FF-LBG and M-FF-LBGAa, the results obtained with the different initialization strategies for N≤64 are very similar. For N=256 with M-FF-LBG, it was possible to obtain a PSNR gain of 0.17 dB by using the MH, MS and All strategies in substitution of the Random initialization, while with M-FF-LBGa, a PSNR gain of 0.12 dB is obtained by the substitution of the Random initialization by MS strategy. For N=512 with the MHS strategy, the M-FF-LBG obtained an average PSNR of 33.08 dB and the M-FF-LBGa obtained 32.89 dB. Comparing these results with the results obtained with Random initialization, gains in terms of PSNR of 0.30 dB and 0.12 dB are observed for M-FF-LBG and M-FF-LBGa, respectively.

Table 17 presents the results of the Friedman test for M-FF-LBG and M-FF-LBGa considering the Peppers image. The results indicate the existence of significant statistical difference between the initialization strategies for N≥128, considering the M-FF-LBG and for N=32 and N≥128 considering the M-FF-LBGa.

**Figure 13:**
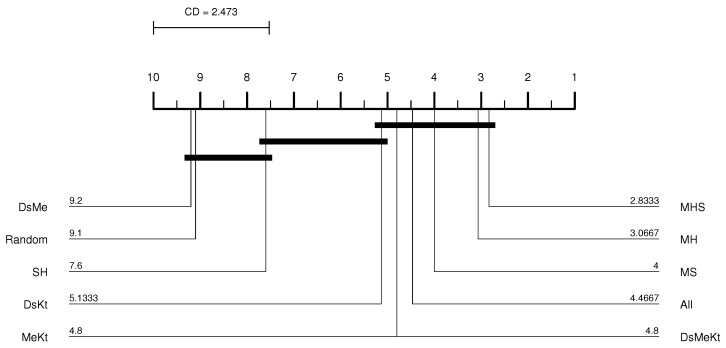
CD diagram of the Peppers image considering the average PSNR (N=512 and M-FF-LBG).

**Figure 14:**
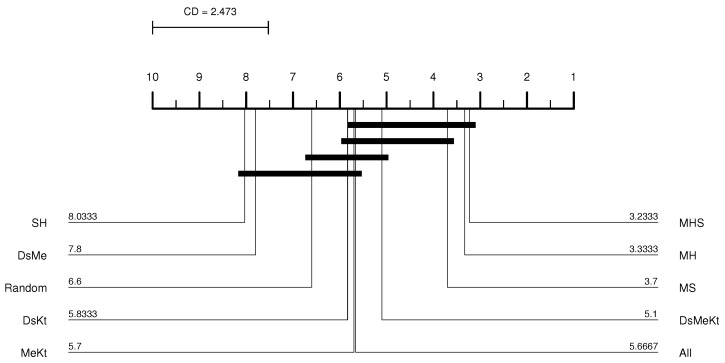
CD diagram of the Peppers image considering the average PSNR (N=512 and M-FF-LBGa).

Figure 13 and Figure 14 present the CD diagram for N=512 with M-FF-LBG and M-FF-LBGa, respectively, considering the Peppers image. Figure 13 indicates that the MHS strategy is the best positioned in the ranking and that it presents statistical superiority only in relation to the SH, Random and DsMe strategies. For the accelerated version of M-FF-LBG (Figure 14), the MHS strategy is also the best positioned in the ranking and presents statistical superiority only in relation to the SH, Random, DsMe and DsKt strategies.

Regarding the results of the average number of iterations, presented in Table 14 and Table 15 for the Peppers image, the superiority of M-FF-LBGa over M-FF-LBG for all *N* is observed, except for N=64 with the strategies DsKt and DsMeKt. For N=256, M-FF-LBG obtained an average number of 43.83 iterations with the MeKt strategy and M-FF-LBGa obtained 34.43 iterations. For N=512, M-FF-LBGa presents an average number of iterations of 48.10 with the DsMeKt strategy, while M-FF-LBGa presents an average number of iterations of 24.20. In particular, for N=512, the substitution of Random initialization by MS strategy results in a reduction of approximately 21% in the average number of iterations considering M-FF-LBG. Considering the accelerated version of M-FF-LBG, for N=512, the substitution of Random initialization by DsMeKt strategy results in a reduction of approximately 46% in the average number of iterations.

**Figure 15:**
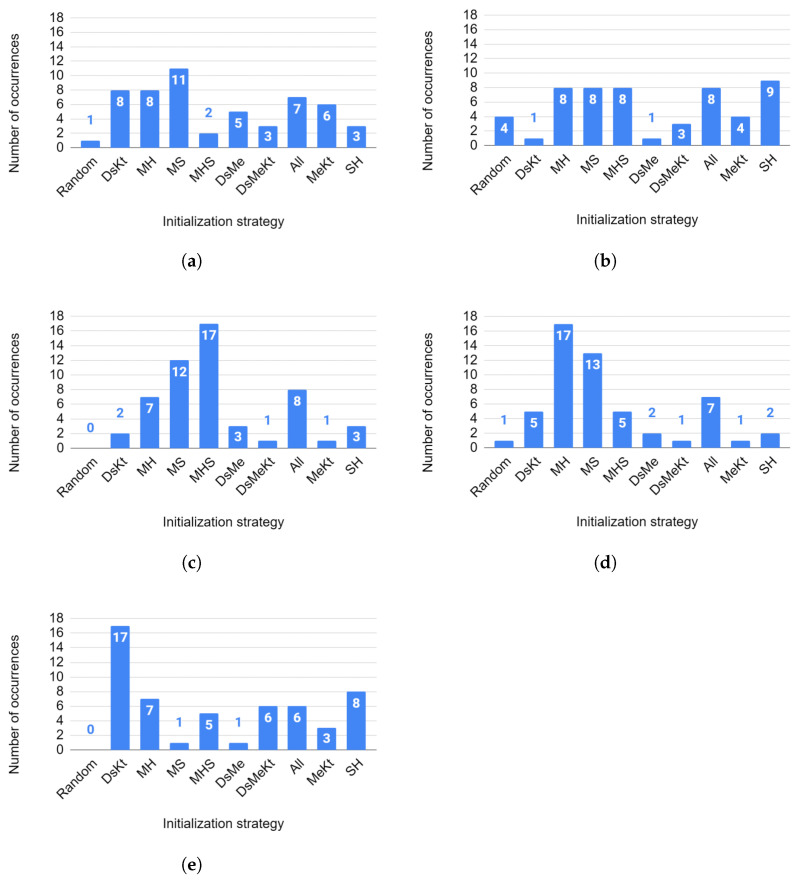
Histogram of the ranking of initialization strategies for the average PSNR for sizes (**a**) N=32, (**b**) N=64, (**c**) N=128, (**d**) N=256 and (**e**) N=512.

To evaluate, in general, the initialization strategies that presented the highest performance, regardless of the image and the algorithm used, we constructed a histogram (for each size *N*) of the ranking of the initialization strategies based on the results obtained with the Nemenyi test for the average PSNR. Figure 15 shows the obtained histograms.

In Figure 15a, for N=32, the MS strategy was 11 times the one that presented the best average PSNR performance. In second position, one observes the DsKt and MH strategies with eight occurrences. For N=64 (Figure 15b), the SH strategy ranked first in the average rank, with nine occurrences. The second place, with eight occurrences, was occupied by all strategies that use the Ma initialization.

In Figure 15c, for N=128, in first place in the average classification, with 17 occurrences, one has the MHS strategy. In second place, with 12 occurrences, one has the MS strategy. For N=256 (Figure 15d), in first place in the average ranking, with 17 occurrences, one has the MH strategy. In second place, with 13 occurrences, one has the MS strategy.

Finally, for N=512 (Figure 15e), the DsKt strategy occupies first place in the ranking with 17 occurrences, while in second position, with 8 occurrences, one has the SH strategy.

It is worth noting that for none of the codebook sizes N=128 and N=512, did the random initialization strategy rank first in the Nemenyi test ranking. For N=32 and N=256 the random initialization ranked first in the Nemenyi test, ranking only once, and for N=64 the random initialization ranked first in the Nemenyi test, ranking four times. This shows that the initialization strategies allowed us to obtain codebooks with higher quality than those obtained by random initialization, thus contributing to obtaining reconstructed images with better quality in terms of PSNR.

**Figure 16:**
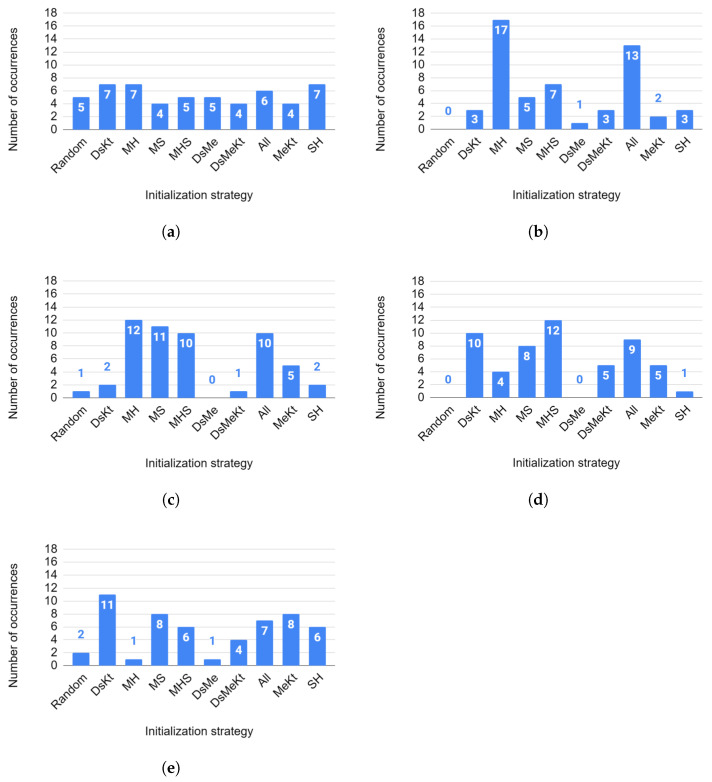
Histogram of the ranking of initialization strategies for the average number of iterations for sizes (**a**) N=32, (**b**) N=64, (**c**) N=128, (**d**) N=256 and (**e**) N=512.

Regarding the average number of iterations, Figure 16 presents the histogram (for each size *N*) of the ranking of initialization strategies based on the results obtained with the Nemenyi test for the average number of iterations.

In Figure 16a, for N=32, a well-distributed histogram is observed in which all initialization strategies were ranked first at least four times. The DsKt, MH and SH strategies occupied the first place in the ranking, with seven occurrences each. The second place, with six occurrences, was occupied by the All strategy.

For N=64, in Figure 16b, the first place was occupied by the MH strategy, with 17 occurrences, and the second place by the All strategy, with 13 occurrences. It is observed that the first to fourth places in the ranking were occupied by all strategies that use the M initialization. Below the fourth position there is a smaller number of occurrences equal to 3. There were no occurrences for the Random strategy.

For N=128 (Figure 16c), a similar behavior was observed. First to third place in the rankings were occupied by all strategies that use the M strategy. The MH strategy occupies first place in the ranking with 12 occurrences, while in second position, with 11 occurrences, one has the MS strategy. In third place there was a tie between the MHS and All strategies, with 10 occurrences each. Below the third position there was a smaller number of occurrences equal to 5. It is also worth highlighting that the random initialization ranked first only once in the Nemenyi test ranking.

In Figure 16d, for N=256, the first place was occupied by the MHS strategy, with 12 occurrences, and second place by the DsKt strategy, with 10 occurrences. There were no occurrences for the Random strategy.

For N=512 (Figure 16e), the first place was occupied by the DsKt strategy, with 11 occurrences. In second place there was a tie between the MS and MeKt strategies, with eight occurrences each.

It is worth mentioning that for codebook sizes N=32,128 and 512, the random initialization strategy ranked first in the Nemenyi test classification, five, one and two times, respectively. The best ranked initialization strategies in the Nemenyi test classification were the strategies that use M initialization. These results indicate that the combination of initialization strategies allows the reduction in the number of algorithm iterations for codebook design, leading to a reduction in the convergence speed of these algorithms.

## 7. Conclusions

Vector quantization (VQ) has been used in signal processing applications such as signal compression. The performance of VQ-based signal processing systems depends on the designed codebooks.

Swarm techniques in conjunction with the LBG algorithm have been proposed as alternatives for codebook design. The quality of the codebooks designed by these algorithms depends on initialization, as they start from initial codebooks that are improved iteratively. Therefore, the initial codebook has a great impact on both the algorithm’s convergence speed and the quality of the reconstructed signals.

This work evaluates different initialization strategies for swarm algorithms combined with LBG. Each initialization strategy consists of combining literature techniques with random initialization. Nine initialization strategies are presented, which are compared with random initialization. Initialization strategies were evaluated on the following algorithms for codebook design: M-FA-LBG, M-PSO-LBG, M-FSS-LBG and their accelerated versions (M-FA-LBGa, M-PSO-LBGa and M-FSS-LBGa).

Evaluations were made in terms of the quality of the reconstructed images evaluated in terms of average PSNR and in terms of the convergence speed from the average number of iterations. Additionally, the Friedman and Nemenyi tests were used to determine whether the results are statistically significantly different.

The results obtained indicate that the initialization strategies provided an increase in the PSNR of the reconstructed images compared to Random initialization. Simulation results reveal gains, in terms average PSNR of reconstructed images, up to 4.43 dB, for Clock with M-PSO-LBG codebooks with size N=512, by using initialization strategies in substitution to Random initialization. We constructed a histogram, for each codebook size *N*, of the ranking of initialization strategies based on the results obtained with the Nemenyi test for the average PSNR. Random initialization ranked first in the Nemenyi test rankings only once for N=32 and N=256, four times for N=64 and not once for N=128 and N=512.

As for the convergence speed, initialization strategies provided savings in the average number of iterations. Simulation results show time savings up to 67.05% for image Clock, with M-FF-LBGa codebooks with size N=512, by using initialization strategies in substitution to Random initialization.

As for future work, we can highlight the evaluation of other initialization strategies different from those used, the proposition of new initialization techniques for swarm algorithms applied to codebook design and the evaluation of the use of the proposed initialization strategies applied to image segmentation and 3D point cloud compression.

## Figures and Tables

**Table 1:** Parameters used in the algorithms.

Parameters	M-FA-LBG and M-FA-LBGa
α	0.7
$\beta_{0}$	0.4
γ	0.001
**Parameters**	**M-PSO-LBG and M-PSO-LBGa**
$c_{1}$	0.8
$c_{2}$	0.2
ω	1.0
**Parameters**	**M-FSS-LBG and M-FSS-LBGa**
initial weight	2500
limit weight	5000
$\alpha_{\mathrm{ind}}$	0.01
$\alpha_{\mathrm{vol}}$	1

**Table 2:** Composition of the initialization techniques used.

Acronym	Random	DSICS	MEIM	KATSA	MA	S.B.	HAD.
Random	10	-	-	-	-	-	-
DsKt	8	1	-	1	-	-	-
MH	8	-	-	-	1		1
MS	8	-	-	-	1	1	-
MHS	7	-	-	-	1	1	1
DsMe	8	1	1	-	-	-	-
DsMeKt	7	1	1	1	-	-	-
All	4	1	1	1	1	1	1
MeKt	8	-	1	1	-	-	-
SH	8	-	-	-		1	1

**Table 3:** Average PSNR results in dB for Clock, Elaine and Peppers images with the M-PSO-LBG algorithm. Bold text is used to highlight the best results for each *N*.

Clock					
**Initialization**	N=32	N=64	N=128	N=256	N=512
Random	26.71	27.93	29.17	30.45	31.20
DsKt	26.83	28.17	29.63	**31.94**	**35.63**
MH	26.77	28.06	29.55	31.30	33.24
MS	26.77	28.02	29.53	31.24	35.34
MHS	26.78	28.07	29.57	31.26	35.37
DsMe	26.71	27.91	29.17	30.41	31.15
DsMeKt	26.83	28.12	29.64	31.72	35.58
All	26.79	28.12	29.62	31.72	35.57
MeKt	**26.84**	**28.19**	**29.67**	**31.94**	**35.63**
SH	26.69	27.89	29.35	31.79	35.41
**Elaine**					
**Initialization**	N=32	N=64	N=128	N=256	N=512
Random	27.83	29.13	30.52	31.88	32.75
DsKt	27.83	29.14	30.48	31.98	33.99
MH	27.83	**29.17**	30.52	**32.08**	33.72
MS	**27.84**	**29.17**	**30.54**	32.03	33.72
MHS	27.82	29.16	30.50	32.01	33.65
DsMe	27.83	29.15	30.51	31.92	32.89
DsMeKt	27.83	29.13	30.46	31.94	34.04
All	27.82	29.15	30.51	32.03	33.98
MeKt	27.82	29.13	30.49	32.01	**34.07**
SH	27.83	29.15	30.51	31.84	33.54
**Peppers**					
**Initialization**	N=32	N=64	N=128	N=256	N=512
Random	26.17	27.48	28.80	30.45	32.69
DsKt	26.17	27.46	28.78	30.52	**33.01**
MH	26.18	27.44	**28.87**	**30.60**	32.70
MS	26.19	27.47	28.86	**30.60**	32.75
MHS	26.18	27.42	28.84	30.56	32.68
DsMe	26.18	27.46	28.79	30.42	32.67
DsMeKt	26.18	27.44	28.75	30.50	32.96
All	26.19	27.43	28.83	30.57	32.94
MeKt	26.18	27.46	28.76	30.53	33.00
SH	**26.20**	**27.49**	28.80	30.43	32.80

**Table 4:** Friedman test for the Clock image with M-PSO-LBG, M-FSS-LBG and M-FSS-LBGa algorithm, considering the average PSNR results.

M-PSO-LBG					
N	**32**	**64**	**128**	**256**	**512**
p **-value**	$2.30\times 10^{-11}$	$5.74\times 10^{-33}$	$1.02\times 10^{-37}$	$7.00\times 10^{-47}$	$2.84\times 10^{-49}$
**M-FSS-LBG**					
N	**32**	**64**	**128**	**256**	**512**
p **-value**	$2.16\times 10^{-20}$	$3.71\times 10^{-35}$	$1.47\times 10^{-38}$	$1.45\times 10^{-27}$	$2.04\times 10^{-35}$
**M-FSS-LBGa**					
N	**32**	**64**	**128**	**256**	**512**
p **-value**	$9.09\times 10^{-24}$	$1.64\times 10^{-35}$	$5.36\times 10^{-37}$	$4.15\times 10^{-29}$	$3.36\times 10^{-33}$

**Table 5:** Average number of iterations obtained for Clock, Elaine and Peppers images with the M-PSO-LBG algorithm. Bold text is used to highlight the best results for each *N*.

Clock					
**Initialization**	N=32	N=64	N=128	N=256	N=512
Random	53.30	58.47	72.13	58.43	20.13
DsKt	38.80	34.50	23.23	51.53	34.70
MH	42.73	52.83	51.57	66.60	28.47
MS	42.60	50.67	56.40	57.80	50.70
MHS	**38.37**	53.03	58.80	57.33	53.13
DsMe	47.77	55.57	74.57	52.93	**19.00**
DsMeKt	40.53	**33.60**	22.87	28.93	33.37
All	45.27	34.47	**21.40**	**28.30**	33.63
MeKt	42.00	38.37	26.87	51.40	39.03
SH	50.50	55.97	53.30	49.73	56.40
**Elaine**					
**Initialization**	N=32	N=64	N=128	N=256	N=512
Random	41.77	45.83	48.27	46.33	**19.00**
DsKt	37.67	41.13	47.63	54.67	38.60
MH	36.60	39.97	32.30	33.67	23.40
MS	36.27	44.93	33.83	30.53	24.20
MHS	31.80	40.40	**29.90**	**26.73**	19.57
DsMe	39.50	42.07	47.23	48.93	22.93
DsMeKt	40.63	41.77	47.10	44.93	45.00
All	31.63	**39.67**	30.23	28.77	38.93
MeKt	38.27	40.23	48.17	53.00	46.30
SH	**27.67**	43.43	50.30	43.10	43.53
**Peppers**					
**Initialization**	N=32	N=64	N=128	N=256	N=512
Random	40.37	42.73	42.07	56.33	68.40
DsKt	38.23	41.53	49.70	49.87	49.20
MH	38.27	33.63	36.73	48.30	**38.90**
MS	43.00	35.03	37.37	48.27	40.13
MHS	**36.83**	**25.67**	35.73	**45.33**	40.40
DsMe	40.90	38.93	45.00	55.77	63.10
DsMeKt	38.03	40.97	48.00	50.53	47.60
All	37.87	26.67	**32.93**	46.03	44.37
MeKt	43.43	39.17	47.40	50.93	45.60
SH	37.53	40.33	46.87	55.37	59.23

**Table 6:** Average PSNR results in dB for Clock, Elaine and Peppers images with the M-PSO-LBGa algorithm. Bold text is used to highlight the best results for each *N*.

Clock					
**Initialization**	N=32	N=64	N=128	N=256	N=512
Random	26.78	27.98	29.29	30.72	32.19
DsKt	**26.86**	**28.18**	29.66	**31.71**	**35.46**
MH	26.83	28.12	29.49	31.30	33.69
MS	26.84	28.08	29.50	31.32	34.98
MHS	26.84	28.11	29.52	31.32	34.98
DsMe	26.80	28.02	29.28	30.70	32.15
DsMeKt	26.85	**28.18**	**29.67**	**31.71**	**35.46**
All	26.83	**28.18**	29.65	**31.71**	**35.46**
MeKt	26.84	**28.18**	29.65	**31.71**	**35.46**
SH	26.79	27.96	29.37	31.41	34.98
**Elaine**					
**Initialization**	N=32	N=64	N=128	N=256	N=512
Random	27.84	29.17	30.53	31.99	33.76
DsKt	27.84	29.15	30.51	31.88	33.94
MH	27.84	29.19	**30.54**	**32.04**	33.73
MS	**27.85**	29.18	30.53	32.02	33.72
MHS	27.84	29.19	**30.54**	32.03	33.71
DsMe	27.84	29.16	30.50	31.98	33.85
DsMeKt	27.83	29.16	30.50	31.88	33.95
All	27.84	**29.20**	**30.54**	**32.04**	33.94
MeKt	**27.85**	29.16	30.49	31.88	**33.96**
SH	27.82	29.16	30.49	31.97	33.66
**Peppers**					
**Initialization**	N=32	N=64	N=128	N=256	N=512
Random	26.18	**27.48**	28.80	30.38	32.59
DsKt	26.19	27.46	28.77	30.49	**32.83**
MH	26.19	27.45	28.84	30.43	32.77
MS	**26.20**	27.46	28.84	30.42	32.78
MHS	**26.20**	27.45	**28.85**	30.47	32.79
DsMe	**26.20**	27.46	28.81	30.40	32.57
DsMeKt	26.18	27.47	28.76	**30.50**	**32.83**
All	**26.20**	27.46	28.84	30.44	**32.83**
MeKt	26.19	27.47	28.76	30.47	**32.83**
SH	**26.20**	**27.48**	28.80	30.42	32.64

**Table 7:** Average number of iterations obtained for Clock, Elaine and Peppers images with the M-PSO-LBGa algorithm. Bold text is used to highlight the best results for each *N*.

Clock					
**Initialization**	N=32	N=64	N=128	N=256	N=512
Random	36.97	42.97	58.77	63.30	50.73
DsKt	35.07	18.87	27.57	**18.00**	21.00
MH	34.53	44.27	46.07	55.07	55.30
MS	35.70	44.87	48.07	57.73	**18.00**
MHS	35.03	45.27	47.47	56.60	**18.00**
DsMe	39.57	45.30	59.60	59.23	50.50
DsMeKt	**33.93**	18.83	28.20	**18.00**	21.00
All	36.90	**18.00**	27.47	**18.00**	21.00
MeKt	37.00	**18.00**	**27.10**	**18.00**	21.00
SH	38.37	39.50	39.67	19.60	**18.00**
**Elaine**					
**Initialization**	N=32	N=64	N=128	N=256	N=512
Random	30.37	34.30	37.23	44.10	51.73
DsKt	30.13	**30.07**	41.37	**19.00**	27.57
MH	24.83	33.07	24.83	22.77	20.33
MS	29.30	31.00	**23.90**	19.87	19.20
MHS	24.53	32.80	24.50	21.03	**18.57**
DsMe	29.33	31.10	33.67	43.07	58.37
DsMeKt	28.90	30.53	38.70	**19.00**	28.23
All	24.13	33.53	24.57	21.53	29.17
MeKt	29.23	30.47	37.80	**19.00**	30.17
SH	**17.00**	31.17	29.33	41.30	36.93
**Peppers**					
**Initialization**	N=32	N=64	N=128	N=256	N=512
Random	29.27	31.43	35.60	41.47	53.97
DsKt	29.80	29.90	31.70	51.47	**22.00**
MH	28.63	**20.97**	24.00	27.10	42.20
MS	28.17	23.83	**23.23**	**25.60**	46.40
MHS	29.13	21.77	24.30	29.30	45.27
DsMe	30.23	29.63	35.97	43.80	52.03
DsMeKt	27.97	31.47	30.43	49.63	**22.00**
All	**27.87**	23.43	24.50	27.57	**22.00**
MeKt	31.27	31.07	31.17	48.67	**22.00**
SH	32.33	25.13	29.90	46.50	47.33

**Table 8:** Average PSNR results in dB for Clock, Elaine and Peppers images with the M-FSS-LBG algorithm. Bold text is used to highlight the best results for each *N*.

Clock					
**Initialization**	N=32	N=64	N=128	N=256	N=512
Random	26.69	27.82	29.00	30.36	32.11
DsKt	26.80	28.11	29.55	31.02	34.66
MH	26.79	28.03	29.52	31.20	33.41
MS	**26.90**	**28.24**	**29.80**	**31.47**	34.59
MHS	26.79	28.03	29.50	31.33	34.70
DsMe	26.73	27.83	29.07	30.30	31.98
DsMeKt	26.81	28.15	29.51	31.15	35.09
All	26.83	28.10	29.63	31.31	**35.37**
MeKt	26.80	28.12	29.55	31.10	35.31
SH	26.68	27.87	29.20	31.24	35.04
**Elaine**					
**Initialization**	N=32	N=64	N=128	N=256	N=512
Random	27.79	29.12	30.48	31.93	33.44
DsKt	27.79	29.14	30.48	31.93	**34.01**
MH	**27.83**	**29.16**	30.51	32.04	33.85
MS	27.80	29.15	30.50	32.02	33.89
MHS	27.82	29.15	**30.53**	**32.07**	33.92
DsMe	27.80	29.13	30.48	31.93	33.33
DsMeKt	27.82	29.12	30.45	31.92	34.00
All	27.81	29.14	30.50	32.02	33.96
MeKt	27.80	29.12	30.49	31.89	33.97
SH	27.82	29.10	30.46	32.00	33.70
**Peppers**					
**Initialization**	N=32	N=64	N=128	N=256	N=512
Random	26.16	27.46	28.84	30.39	32.44
DsKt	26.16	27.46	28.83	30.44	32.84
MH	26.17	27.45	**28.90**	30.61	33.00
MS	26.16	27.45	**28.90**	30.60	32.93
MHS	26.17	27.45	28.89	30.59	32.92
DsMe	**26.18**	27.46	28.83	30.41	32.38
DsMeKt	26.16	**27.47**	28.84	30.42	32.84
All	26.17	**27.47**	28.88	**30.68**	**33.04**
MeKt	26.17	27.43	28.80	30.44	32.86
SH	26.16	27.46	28.83	30.40	32.87

**Table 9:** Average PSNR results in dB for Clock, Elaine and Peppers images with the M-FSS-LBGa algorithm. Bold text is used to highlight the best results for each *N*.

Clock					
**Initialization**	N=32	N=64	N=128	N=256	N=512
Random	26.71	27.81	29.05	30.40	32.09
DsKt	26.82	28.10	29.53	31.07	35.26
MH	26.78	28.03	29.51	31.19	33.59
MS	**26.91**	**28.23**	**29.79**	**31.50**	34.69
MHS	26.77	28.03	29.54	31.33	34.48
DsMe	26.71	27.84	29.03	30.38	31.96
DsMeKt	26.81	28.11	29.52	31.04	34.62
All	26.85	28.10	29.54	31.44	**35.27**
MeKt	26.80	28.14	29.52	31.10	34.89
SH	26.69	27.83	29.22	31.32	35.11
**Elaine**					
**Initialization**	N=32	N=64	N=128	N=256	N=512
Random	27.81	29.12	30.46	31.92	33.38
DsKt	27.79	29.11	30.46	31.95	33.94
MH	**27.83**	**29.17**	**30.52**	32.05	33.86
MS	27.81	**29.17**	**30.52**	**32.07**	33.95
MHS	27.81	29.16	**30.52**	32.01	33.94
DsMe	27.80	29.13	30.46	31.96	33.51
DsMeKt	27.81	29.13	30.47	31.91	**34.02**
All	**27.83**	29.16	**30.52**	32.04	33.97
MeKt	27.81	29.12	30.49	31.92	34.00
SH	27.82	29.11	30.44	31.96	33.80
**Peppers**					
**Initialization**	N=32	N=64	N=128	N=256	N=512
Random	26.16	**27.48**	28.81	30.44	32.35
DsKt	**26.17**	**27.48**	28.84	30.42	32.76
MH	**26.17**	27.46	28.90	**30.63**	32.99
MS	**26.17**	27.47	28.86	30.62	32.93
MHS	**26.17**	27.44	28.88	30.61	32.98
DsMe	**26.17**	27.46	28.84	30.41	32.42
DsMeKt	26.16	**27.48**	28.81	30.42	32.90
All	**26.17**	27.46	**28.92**	30.62	**33.08**
MeKt	**26.17**	27.47	28.83	30.41	32.87
SH	**26.17**	27.44	28.82	30.39	32.85

**Table 10:** Average number of iterations obtained for Clock, Elaine and Peppers images with the M-FSS-LBG algorithm. Bold text is used to highlight the best results for each *N*.

Clock					
**Initialization**	N=32	N=64	N=128	N=256	N=512
Random	25.43	26.80	29.90	30.30	30.93
DsKt	21.73	24.73	29.23	28.43	18.77
MH	22.40	**23.13**	26.87	25.40	20.83
MS	21.17	25.20	29.27	23.40	16.00
MHS	21.27	24.13	**24.80**	17.63	17.60
DsMe	25.90	27.57	32.33	28.13	27.20
DsMeKt	22.77	28.13	27.83	32.33	15.27
All	**19.70**	24.10	26.53	19.10	13.63
MeKt	23.03	27.63	30.17	30.40	**12.00**
SH	25.53	28.30	28.87	**17.10**	16.83
**Elaine**					
**Initialization**	N=32	N=64	N=128	N=256	N=512
Random	15.47	18.27	23.87	29.37	29.53
DsKt	**13.77**	19.83	22.07	28.40	16.67
MH	17.13	17.10	15.03	15.37	17.90
MS	16.07	**16.03**	15.13	14.97	19.37
MHS	18.00	16.90	16.50	17.80	18.63
DsMe	15.63	18.33	24.03	30.90	26.10
DsMeKt	16.77	19.10	21.27	28.33	16.53
All	17.47	16.10	**14.73**	**14.67**	17.27
MeKt	16.00	17.60	23.80	26.20	**16.13**
SH	18.67	16.80	23.47	28.10	29.67
**Peppers**					
**Initialization**	N=32	N=64	N=128	N=256	N=512
Random	15.93	19.57	26.33	31.87	37.60
DsKt	15.73	20.07	24.67	31.50	28.67
MH	16.43	**17.07**	21.13	26.27	32.37
MS	15.83	17.13	22.13	25.63	29.63
MHS	17.20	17.77	**19.27**	**25.13**	29.73
DsMe	16.63	18.80	26.97	32.00	34.23
DsMeKt	16.90	20.30	27.33	29.97	29.67
All	17.50	18.53	20.00	28.17	**27.27**
MeKt	15.93	17.80	23.67	31.57	27.73
SH	**15.60**	20.03	26.27	33.07	31.97

**Table 11:** Average number of iterations obtained for Clock, Elaine and Peppers images with the M-FSS-LBGa algorithm. Bold text is used to highlight the best results for each *N*.

Clock					
**Initialization**	N=32	N=64	N=128	N=256	N=512
Random	25.47	27.60	31.33	31.67	30.80
DsKt	22.83	27.30	28.37	29.70	13.37
MH	**20.27**	23.50	26.00	26.13	25.83
MS	21.77	25.43	29.63	23.70	20.53
MHS	21.67	23.87	27.13	**21.10**	21.20
DsMe	25.13	26.87	30.83	30.23	28.37
DsMeKt	23.10	27.23	27.67	28.17	15.43
All	21.27	**22.80**	**22.47**	23.77	**13.23**
MeKt	21.93	27.73	27.93	29.87	14.90
SH	26.00	27.43	28.13	21.87	19.97
**Elaine**					
**Initialization**	N=32	N=64	N=128	N=256	N=512
Random	15.90	17.67	22.63	29.77	27.13
DsKt	**15.47**	17.67	21.73	29.70	**14.43**
MH	18.13	17.60	16.20	16.10	18.70
MS	16.10	17.77	16.30	17.50	20.97
MHS	16.17	**16.60**	**15.73**	**14.50**	21.90
DsMe	15.57	19.20	22.70	30.57	31.37
DsMeKt	15.77	19.90	22.83	26.57	17.43
All	18.40	17.13	15.97	16.33	16.20
MeKt	15.83	18.83	23.10	28.10	15.83
SH	18.57	17.57	21.57	26.13	32.53
**Peppers**					
**Initialization**	N=32	N=64	N=128	N=256	N=512
Random	16.87	21.60	25.00	33.70	33.90
DsKt	**16.13**	20.67	25.07	30.80	29.57
MH	16.23	18.03	21.30	27.93	30.87
MS	17.07	18.60	**17.93**	27.40	30.77
MHS	16.73	**16.33**	19.10	26.23	30.87
DsMe	16.93	19.27	26.70	32.00	35.07
DsMeKt	16.27	21.07	24.40	31.37	31.17
All	16.97	18.50	22.57	**26.10**	29.83
MeKt	16.90	20.20	26.43	29.47	**29.27**
SH	17.40	18.73	24.37	32.03	30.73

**Table 12:** Average PSNR results in dB for Clock, Elaine and Peppers images with the M-FF-LBG algorithm. Bold text is used to highlight the best results for each *N*.

Clock					
**Initialization**	N=32	N=64	N=128	N=256	N=512
Random	26.71	27.89	29.19	30.75	32.58
DsKt	26.87	28.27	**29.89**	**32.13**	35.67
MH	26.80	28.08	29.66	31.51	34.04
MS	26.82	28.13	29.80	31.80	35.52
MHS	26.81	28.08	29.68	31.65	35.53
DsMe	26.72	27.89	29.19	30.73	32.56
DsMeKt	**26.88**	28.28	**29.89**	32.11	**35.69**
All	26.82	**28.29**	29.86	32.09	35.66
MeKt	26.87	28.28	**29.89**	32.12	35.67
SH	26.67	27.88	29.47	32.00	35.53
**Elaine**					
**Initialization**	N=32	N=64	N=128	N=256	N=512
Random	27.83	29.17	30.56	32.07	33.86
DsKt	27.83	29.17	30.61	32.17	**34.22**
MH	**27.85**	29.21	**30.64**	32.24	34.15
MS	27.84	29.21	30.63	32.25	34.15
MHS	**27.85**	**29.22**	**30.64**	32.25	34.15
DsMe	27.83	29.18	30.55	32.06	33.87
DsMeKt	27.82	29.17	30.60	32.17	**34.22**
All	**27.85**	29.21	**30.64**	**32.27**	**34.22**
MeKt	27.84	29.17	30.60	32.17	**34.22**
SH	27.83	29.18	30.57	32.13	33.86
**Peppers**					
**Initialization**	N=32	N=64	N=128	N=256	N=512
Random	26.19	27.51	28.88	30.54	32.78
DsKt	26.19	27.51	28.87	30.63	33.06
MH	**26.21**	27.52	**28.98**	**30.71**	33.05
MS	26.20	27.52	28.97	**30.71**	32.94
MHS	**26.21**	27.52	**28.98**	30.70	**33.08**
DsMe	26.19	27.50	28.87	30.55	32.78
DsMeKt	26.19	27.50	28.87	30.63	33.07
All	**26.21**	27.52	**28.98**	**30.71**	33.07
MeKt	26.19	27.51	28.88	30.63	33.07
SH	26.20	**27.53**	28.88	30.54	32.94

**Table 13:** Average PSNR results in dB for Clock, Elaine and Peppers images with the M-FF-LBGa algorithm. Bold text is used to highlight the best results for each *N*.

Clock					
**Initialization**	N=32	N=64	N=128	N=256	N=512
Random	26.81	28.14	29.56	31.29	32.99
DsKt	26.88	28.19	29.65	**31.81**	35.49
MH	26.86	28.17	29.73	31.50	33.87
MS	26.85	28.20	**29.76**	31.61	34.92
MHS	26.85	28.16	29.70	31.49	34.94
DsMe	26.81	28.14	29.54	31.28	32.90
DsMeKt	**26.89**	28.20	29.63	**31.81**	35.49
All	26.85	28.20	29.64	31.78	**35.50**
MeKt	26.87	**28.21**	29.64	31.79	35.49
SH	26.81	28.11	29.59	31.53	34.93
**Elaine**					
**Initialization**	N=32	N=64	N=128	N=256	N=512
Random	27.86	29.23	30.67	**32.20**	34.09
DsKt	27.86	29.24	30.64	32.10	34.02
MH	**27.88**	29.26	30.65	32.15	34.06
MS	27.86	29.25	30.67	32.16	34.04
MHS	**27.88**	**29.27**	30.65	32.14	33.98
DsMe	27.86	29.24	**30.68**	32.19	**34.10**
DsMeKt	27.87	29.23	30.62	32.10	34.02
All	27.87	29.25	30.65	32.13	34.02
MeKt	27.87	29.23	30.63	32.08	34.01
SH	27.85	29.24	**30.68**	32.18	33.78
**Peppers**					
**Initialization**	N=32	N=64	N=128	N=256	N=512
Random	26.20	27.56	28.93	30.48	32.77
DsKt	26.20	**27.57**	28.85	30.43	32.84
MH	**26.22**	27.56	**28.95**	30.59	32.87
MS	**26.22**	27.56	**28.95**	**30.60**	32.88
MHS	**26.22**	27.55	28.94	30.56	**32.89**
DsMe	26.20	**27.57**	28.93	30.49	32.72
DsMeKt	26.20	**27.57**	28.86	30.42	32.85
All	**26.22**	27.56	28.93	30.59	32.84
MeKt	26.21	**27.57**	28.85	30.43	32.84
SH	26.20	**27.57**	28.93	30.54	32.66

**Table 14:** Average number of iterations obtained for Clock, Elaine and Peppers images with the M-FF-LBG algorithm. Bold text is used to highlight the best results for each *N*.

Clock					
**Initialization**	N=32	N=64	N=128	N=256	N=512
Random	65.40	48.13	46.93	48.93	45.50
DsKt	**38.37**	38.37	**40.43**	**42.90**	36.17
MH	46.40	46.03	46.70	50.30	48.17
MS	44.73	46.03	48.20	53.07	52.43
MHS	46.53	43.27	46.33	53.70	54.80
DsMe	68.60	47.83	48.40	49.03	47.97
DsMeKt	44.13	38.13	41.30	43.93	35.13
All	43.07	**36.93**	40.90	46.43	**32.20**
MeKt	43.70	37.13	41.97	44.80	38.17
SH	64.00	45.57	49.07	49.90	53.37
**Elaine**					
**Initialization**	N=32	N=64	N=128	N=256	N=512
Random	43.83	50.27	51.10	49.73	45.83
DsKt	44.20	49.33	52.77	48.13	53.90
MH	43.13	**47.67**	42.80	**42.70**	**43.87**
MS	40.30	52.33	**41.17**	45.10	47.80
MHS	40.23	53.63	43.77	44.10	44.80
DsMe	42.10	51.83	53.43	49.07	48.00
DsMeKt	40.70	50.83	47.13	48.33	52.20
All	40.17	49.53	44.13	46.77	52.73
MeKt	45.67	50.37	48.77	48.17	52.73
SH	**39.00**	56.00	53.70	47.43	50.00
**Peppers**					
**Initialization**	N=32	N=64	N=128	N=256	N=512
Random	48.40	48.47	45.87	51.27	55.07
DsKt	45.07	44.57	41.97	**42.00**	47.40
MH	50.37	**37.80**	40.70	46.53	51.17
MS	43.83	38.50	39.57	46.00	**43.67**
MHS	45.20	42.77	**39.13**	48.60	51.97
DsMe	47.00	48.10	48.10	52.37	52.30
DsMeKt	42.73	43.77	42.53	42.43	48.10
All	45.37	38.50	42.53	47.10	51.07
MeKt	**42.50**	46.97	43.60	43.83	46.33
SH	42.73	43.50	45.10	48.53	51.13

**Table 15:** Average number of iterations obtained for Clock, Elaine and Peppers images with the M-FF-LBGa algorithm. Bold text is used to highlight the best results for each *N*.

Clock					
**Initialization**	N=32	N=64	N=128	N=256	N=512
Random	35.63	47.33	49.97	55.63	50.07
DsKt	35.43	23.57	19.57	37.30	22.60
MH	35.10	41.63	40.27	43.17	37.73
MS	34.63	43.10	39.73	38.57	17.70
MHS	35.37	39.60	39.57	34.33	**16.50**
DsMe	39.07	49.37	48.20	55.67	48.60
DsMeKt	35.40	**22.43**	18.77	34.77	22.57
All	**32.03**	25.27	19.63	32.43	21.37
MeKt	33.80	24.23	**18.27**	32.03	21.23
SH	43.60	41.07	42.80	**22.40**	16.87
**Elaine**					
**Initialization**	N=32	N=64	N=128	N=256	N=512
Random	36.67	43.60	43.17	47.20	51.50
DsKt	35.33	43.43	45.33	44.13	**36.73**
MH	33.23	40.33	38.10	35.07	41.97
MS	31.27	**40.23**	40.37	37.57	44.13
MHS	29.73	41.03	**37.53**	**34.00**	40.23
DsMe	36.83	45.27	43.20	47.67	52.47
DsMeKt	37.10	41.73	42.97	44.60	38.20
All	32.77	42.97	39.33	35.73	41.50
MeKt	35.13	41.77	43.00	42.90	38.20
SH	**28.23**	42.70	43.50	45.20	38.07
**Peppers**					
**Initialization**	N=32	N=64	N=128	N=256	N=512
Random	34.47	39.20	45.87	37.57	44.73
DsKt	34.87	44.73	37.73	34.93	25.47
MH	35.60	36.90	34.00	40.10	39.17
MS	36.93	36.33	35.07	36.10	40.80
MHS	36.57	**36.23**	35.90	35.93	41.03
DsMe	33.57	41.90	39.33	39.50	40.87
DsMeKt	35.60	44.33	38.17	36.73	**24.20**
All	36.27	36.37	**33.60**	37.77	26.00
MeKt	35.90	46.33	37.03	**34.43**	24.47
SH	**30.77**	41.47	40.60	41.23	38.73

**Table 16:** Friedman test for the Elaine image with M-FF-LBG and M-FF-LBGa algorithm, considering the average PSNR results.

M-FF-LBG					
N	**32**	**64**	**128**	**256**	**512**
p **-value**	$5.33\times 10^{-03}$	$5.14\times 10^{-14}$	$6.67\times 10^{-26}$	$1.33\times 10^{-45}$	$6.24\times 10^{-30}$
**M-FF-LBGa**					
N	**32**	**64**	**128**	**256**	**512**
p **-value**	$1.02\times 10^{-02}$	$2.92\times 10^{-07}$	$8.25\times 10^{-06}$	$4.05\times 10^{-06}$	$5.69\times 10^{-03}$

**Table 17:** Friedman test for the Peppers image with M-PSO-LBGa, M-FSS-LBG, M-FSS-LBGa, M-FF-LBG and M-FF-LBGa algorithm, considering the average PSNR results.

M-PSO-LBGa					
N	**32**	**64**	**128**	**256**	**512**
p **-value**	$4.49\times 10^{-01}$	$8.13\times 10^{-01}$	$9.43\times 10^{-09}$	$4.64\times 10^{-06}$	$3.09\times 10^{-25}$
**M-FSS-LBG**					
N	**32**	**64**	**128**	**256**	**512**
p **-value**	$2.42\times 10^{-01}$	$5.75\times 10^{-01}$	$3.44\times 10^{-06}$	$1.13\times 10^{-20}$	$4.20\times 10^{-19}$
**M-FSS-LBGa**					
N	**32**	**64**	**128**	**256**	**512**
p **-value**	$9.49\times 10^{-01}$	$1.21\times 10^{-01}$	$1.54\times 10^{-09}$	$6.16\times 10^{-20}$	$3.77\times 10^{-18}$
**M-FF-LBG**					
N	**32**	**64**	**128**	**256**	**512**
p **-value**	$7.54\times 10^{-02}$	$1.70\times 10^{-01}$	$3.77\times 10^{-37}$	$1.09\times 10^{-42}$	$1.31\times 10^{-29}$
**M-FF-LBGa**					
N	**32**	**64**	**128**	**256**	**512**
p **-value**	$8.54\times 10^{-03}$	$4.00\times 10^{-01}$	$1.63\times 10^{-10}$	$3.25\times 10^{-16}$	$9.54\times 10^{-15}$

**Table 18:** Friedman test for the Peppers image with the M-PSO-LBGa algorithm, considering the average number of iterations results.

*N*	32	64	128	256	512
p **-value**	$2.96\times 10^{-01}$	$3.65\times 10^{-15}$	$3.24\times 10^{-18}$	$8.87\times 10^{-31}$	$4.51\times 10^{-42}$

## Data Availability

All data used in this paper are acquired from the USC-SIPI Image Database (http://sipi.usc.edu/database/, accessed on 1 February 2024).
